# Outflow Boundary Conditions for Blood Flow in Arterial Trees

**DOI:** 10.1371/journal.pone.0128597

**Published:** 2015-05-22

**Authors:** Tao Du, Dan Hu, David Cai

**Affiliations:** 1 Department of Mathematics, Institute of Natural Sciences, and MOE-LSC, Shanghai Jiao Tong University, Shanghai, China; 2 Courant Institute of Mathematical Sciences and Center for Neural Science, New York University, New York, U.S.A.; 3 NYUAD Institute, New York University Abu Dhabi, Abu Dhabi, UAE; University of Arizona, UNITED STATES

## Abstract

In the modeling of the pulse wave in the systemic arterial tree, it is necessary to truncate small arterial crowns representing the networks of small arteries and arterioles. Appropriate boundary conditions at the truncation points are required to represent wave reflection effects of the truncated arterial crowns. In this work, we provide a systematic method to extract parameters of the three-element Windkessel model from the impedance of a truncated arterial tree or from experimental measurements of the blood pressure and flow rate at the inlet of the truncated arterial crown. In addition, we propose an improved three-element Windkessel model with a complex capacitance to accurately capture the fundamental-frequency time lag of the reflection wave with respect to the incident wave. Through our numerical simulations of blood flow in a single artery and in a large arterial tree, together with the analysis of the modeling error of the pulse wave in large arteries, we show that both a small truncation radius and the complex capacitance in the improved Windkessel model play an important role in reducing the modeling error, defined as the difference in dynamics induced by the structured tree model and the Windkessel models.

## Introduction

In traditional Chinese and Greek medicine, the temporal profile of the blood pressure is believed to be an important indicator of the state of human body [1, 2]. Information carried by the pulse wave, in particular, the amplitude and the rhythm, has also been used in diagnosis of different cardiovascular diseases such as hypertension, atherosclerosis, and stenosis [3–6]. Physiological experiments and mathematical modeling have been carried out in the study of physiological and mechanic properties related to the blood flow [7–9]. For example, one-dimensional models predicting the blood pressure and flow rate in large arteries have been used to predict pulse wave propagation [10–17].

**Fig 1:**
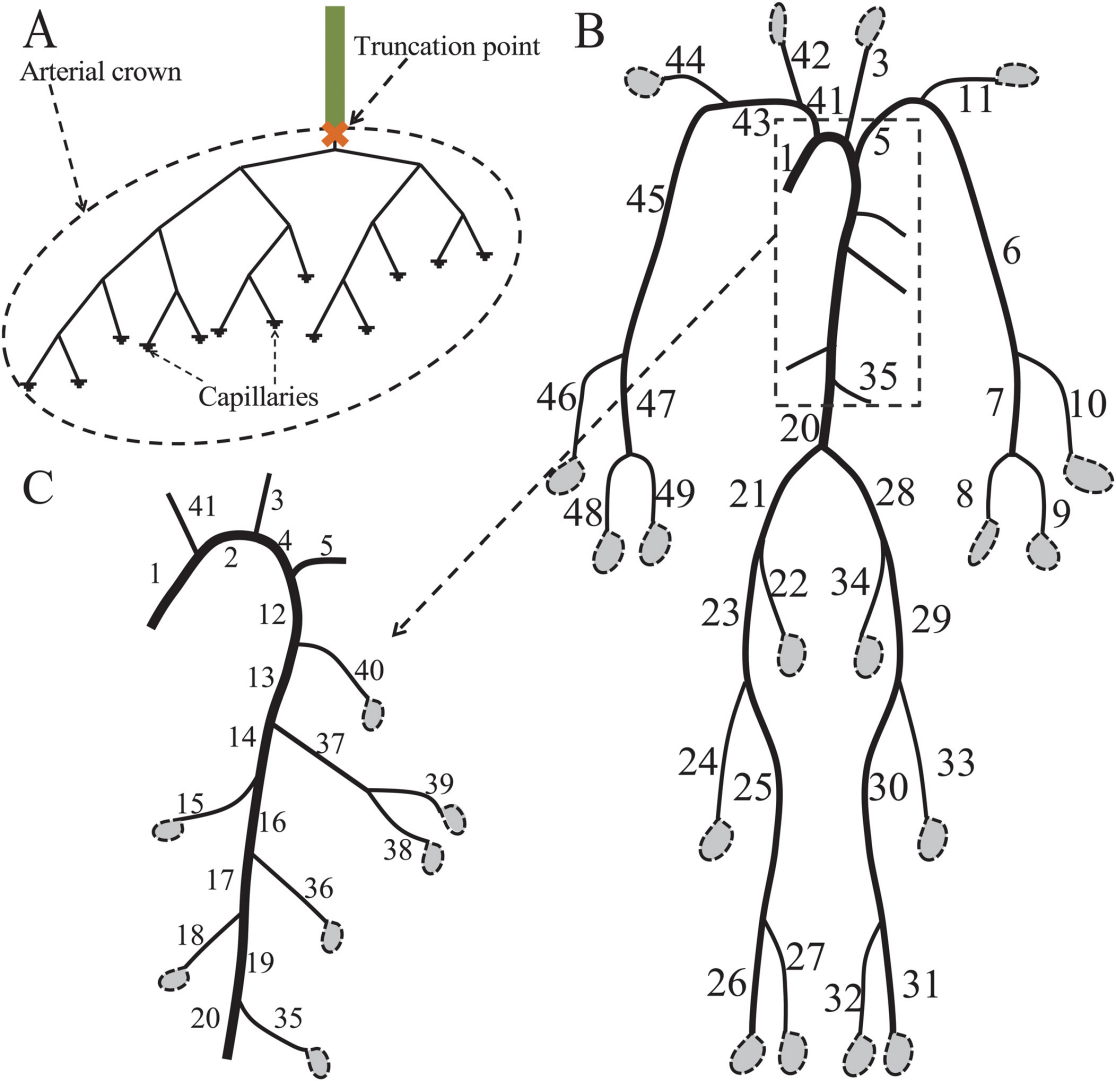
Schematics of a single artery and an arterial tree model. A: a single artery (green tube) with a truncated arterial crown. The outlet of the green vessel is defined as the truncation point. The radius of the root vessel of the truncated arterial crown is defined as truncation radius, which is the same as the radius of the green one. B: the main human systemic arterial tree, based on the data in Ref. [17]. C: details of the principal aortic branches. Each dashed ellipsoid attached to an outlet in B and C represents a truncated arterial crown as exemplified in A.

In general, there is a large amount of small vessels in an arterial tree. To reduce the complexity in the simulation of the blood flow, it is necessary to truncate the small arterial crowns, which are downstream arterial trees from the truncation points (as illustrated in Fig 1A). At the truncation points, suitable outflow boundary conditions for the pulse wave in the large arteries are used to represent wave reflection effects of the truncated arterial crowns. Various types of outflow boundary conditions have been used in the previous works, including the constant resistance model (CR) [18–21], the tapering-vessel model [22], the Windkessel model (WK) [6, 23–26], and the structured tree model (ST) [17, 27–30]. In a number of specialized applications, a non-constant resistance model has been used to model the effect of cerebral autoregulation in the brain [31], and a structured tree model incorporating the effects of geometry, compliance, and respiration has been used to mimic the pulmonary vascular system [32, 33]. It has also been reported that outflow boundary conditions can greatly affect the wave profile in the upstream arteries [11, 28]. Therefore, it is important to systematically investigate the validity of the outflow boundary conditions.

The CR and WK models are obtained through an analogy to electric circuit components. The CR model is represented by a resistor, in which the blood pressure is assumed to be proportional to the blood flow rate. This boundary condition often leads to a large non-physical reflection of the pulse wave because it does not capture the compliance of the downstream arterial walls [11]. In the three-element WK model, there are three physical quantities, namely, the peripheral resistance, the characteristic resistance, and the capacitance [6, 34–37]. The capacitance is introduced to take into account the compliance of the downstream arterial walls [38]. If the parameters of the WK model for the truncated arterial crowns are able to incorporate their resistant and compliant properties, the one-dimensional model of blood flow in the large arteries can capture the profile of the pulse wave [28]. In general, it is difficult to obtain the structures of all the truncated arterial crowns. To circumvent this issue, structured trees are constructed to model the truncated arterial crowns [28]. The impedance of a structured tree, which is the ratio of the Fourier coefficient of the blood pressure to that of the flow rate at the inlet of the structured tree, is obtained from the linearized system of the one-dimensional model of blood flow in the structured tree. With the ST model, detailed characteristics of the pulse wave such as the dicrotic wave are observed in the simulations [11, 29, 30, 32, 33]. Therefore, it is important to carry out a systematic comparison among these models.

In general, it is difficult to obtain all the information of the arterial crowns. In order to obtain an accurate description of pulse wave in a large arterial tree, a number of crucial questions need to be addressed: How to parameterize the simplified models (*e.g.*, the WK model) from the geometrical structure and elastic property of the truncated arterial crown in order to reduce the modeling error of the pulse wave? What controls the modeling error of the pulse wave? Finally, can we improve the WK model for a better description of the effects of the truncated arterial crown? The answers to these questions are important and can help us relate experimental measurements of blood pressure and flow rate to the structural information of arterial trees.

In this work, a systematic procedure is designed to extract proper values of the parameters of the three-element WK model from the impedance of a truncated arterial crown. A theoretical formula is derived for the characteristic resistance, which depends only on the elastic and geometrical properties of the root vessel. The fundamental-frequency impedance is used to determine the capacitance. A complex Windkessel model (CWK), in which the capacitance can be complex, is proposed in order to accurately capture the fundamental-frequency time lag between the blood pressure and flow rate, thus reducing the modeling error of the pulse wave in large arteries. The modeling error of the pulse wave is defined as the relative difference of the blood pressures (or flow rates) in the large arteries between the ST model and the WK (or CWK) model used as the outflow boundary conditions. By using a characteristic time scale for a truncated arterial crown, we can estimate the modeling error and show that the modeling error decreases when the truncation radius (TR) (Fig 1A) becomes small. Furthermore, through our numerical simulations, we show that the complex capacitance in the CWK model can significantly reduce the modeling error of the pulse wave.

## Mathematical Model

### One-dimensional model of blood flow in arteries

One-dimensional models are widely used in studying the blood pressure and flow wave propagation in arterial networks [5, 6, 11, 12, 15]. An artery can be modeled as a cylindrical tube with fixed length, *L*, and radius, *R*(*x*, *t*), where *x* and *t* are the spatial and temporal coordinates, respectively. The dynamics of the cross-sectional area, *A*(*x*, *t*), and the mean blood flow velocity averaged over the cross section, *u*(*x*, *t*), is governed by the following equations obtained from conservation laws of mass and momentum:

$$\begin{array}{c} \frac{\partial }{\partial t}(\begin{array}{c} A\\ u \end{array})+\frac{\partial }{\partial x}(\begin{array}{c} uA\\ \frac{u^{2}}{2}+\frac{p}{\rho } \end{array})=(\begin{array}{c} 0\\ \frac{1}{A}\frac{\partial }{\partial x}(Au^{2}-Aw)+\frac{f}{\rho } \end{array}), \end{array}$$ (1)

where *ρ* is the blood density, *p* is the blood pressure, *w* is the mean square of the velocity averaged over the cross section at *x*, and $f=\frac{2\pi R}{A}\tau$ represents the viscous effect arising from the shear stress, *τ*, on the arterial wall.

There are five variables (*u*, *A*, *f*, *w*, and *p*) but only two equations in Eq (1). We need three more relations to obtain a closed system. First, a velocity profile on a cross section is used to obtain *w* and *f*. The Womersley velocity profile [39] has been shown to be able to capture the boundary layer effect for pulsatile blood flow when the Womersley number, $W=r_{0}\sqrt{\rho \omega /\mu }$, is large [11, 16, 40, 41], where *r*_0_ is the unstressed radius of the vessel when the transmural blood pressure is zero, *ω* is the frequency of the wave, and *μ* = 0.046 *gcm* ^−1^ *s* ^−1^ is the blood viscosity. The Fourier mode of the Womersley velocity profile is given by

$$\begin{array}{c} \hat{v}\left(r,\omega \right)=\{\begin{array}{cc} -\frac{\hat{P_{x}}\left(\omega \right)}{i\omega \rho }[1-\frac{J_{0}(r\sqrt{-i\omega \rho /\mu })}{J_{0}(r_{0}\sqrt{-i\omega \rho /\mu })}] & \;\;\text{for}\;\;\omega \neq 0,\\ -\frac{\hat{P_{x}}\left(0\right)\left(r_{0}^{2}-r^{2}\right)}{4\mu } & \;\;\text{otherwise,}\;\; \end{array} \end{array}$$ (2)

where $\hat{P_{x}}(\omega )$ is the Fourier mode of *p*_*x*_(*t*), *J*_0_(⋅) is the zeroth order Bessel function of the first kind, and *r* is the polar coordinate in the cross section. Because of the boundary layer effect, the viscous effect *f* can be significantly enhanced compared to that obtained from the Poiseuille flow profile [42]. For the Poiseuille flow, the velocity profile in a cross section is parabolic and there is no boundary layer. In the intermediate-sized arteries, the Womersley number is large when the frequency is high. In large arteries, the Womersley number is also large even when the frequency is low. For example, for a vessel with the radius *r*_0_ = 0.5 *cm* and *ω* = 4*ω*_0_, where $\omega_{0}=\frac{2\pi }{T}$ is the fundamental frequency and the period of one heartbeat period *T*, say, 0.75 *s*, the Womersley number is 13.5. Therefore, it is important to use the Womersley model in these vessels to capture the boundary layer effect. Under the Womersley velocity profile, the Fourier mode of the wall shear stress is given by

$$\begin{array}{c} \hat{\tau }\left(\omega \right)=\{\begin{array}{cc} -\frac{i\rho \omega \hat{Q}\left(\omega \right)r_{0}F_{J}}{2A_{0}(1-F_{J})}, & \text{for}\;\;\omega \neq 0,\\ -\frac{4\pi \mu r_{0}}{A_{0}^{2}}\hat{Q}\left(0\right), & \text{otherwise}, \end{array} \end{array}$$ (3)

where $\hat{Q}(\omega )$ is the Fourier mode of the blood flow rate, *q*(*t*), $F_{J}=\frac{2J_{1}(h_{0})}{h_{0}J_{0}(h_{0})}$, *h*_0_ = *i* ^3/2^ *W*, *J*_1_(⋅) is the first order Bessel function of the first kind, and $A_{0}(x)=\pi r_{0}^{2}$ is the unstressed cross-sectional area of the vessel. The periodic velocity profile, *v*(*r*, *t*), and the wall shear stress, *τ*(*t*), can be computed from $\hat{v}(r,\omega )$ and $\hat{\tau }(\omega )$ by the inverse Fourier transform, respectively.

To further close the system, we need a state equation between the blood pressure, *p*, and the cross sectional area, *A* [12, 13]. By approximating the arterial wall as an elastic tissue, the state equation can be derived from the Laplace law,

$$\begin{array}{c} p\left(A\right)=\frac{1}{2}\rho c^{2}[{\left(\frac{A(x,t)}{A_{0}\left(x\right)}\right)}^{2}-1],\;\;\;\;c=\sqrt{\frac{2Eh}{3\rho r_{0}\left(x\right)}}, \end{array}$$ (4)

where *c* is the pulse wave speed in the vessel, *E* is the Young’s Modulus, and *h* is the wall thickness of the arterial wall [11]. Another form of the state equation, $p(A)=\frac{4Eh}{3r_{0}}\left(1-\sqrt{\frac{A_{0}(x)}{A(x,t)}}\right)$, has been also used in the previous works of Refs. [17, 27, 28]. For small deformation, *i.e.*, $\frac{A(x,t)}{A_{0}(x)}-1$ is small, the two state equations are identical to the first order of $\frac{\delta A(x,t)}{A_{0}(x)}=\frac{A(x,t)-A_{0}(x)}{A_{0}(x)}$.

As in Refs. [43, 44], the thickness of the vessel wall, *h*, is assumed to be given by $h=h_{a}{\left(r_{0}/r_{e}\right)}^{h_{b}}$, where the parameters *h*_*a*_ = 0.1204 *cm*, *h*_*b*_ = 0.6244, and *r*_*e*_ = 1.0 *cm* are fitted with the experimental data in Ref. [45]. The Young’s modulus is assumed to satisfy the relation $E=k_{1}e^{k_{2}r_{0}}+k_{3}$ [17], where *k*_1_ = 2.055 × 10^7^ *gcm* ^−1^ *s* ^-2^, *k*_2_ = −5.634 *cm* ^−1^, and *k*_3_ = 4.182 × 10^6^ *gcm* ^−1^ *s* ^-2^ are fitted with experimental data in Ref. [6]. In this work, we neglect the tapering of a single vessel, *i.e.*, *r*_0_(*x*), *A*_0_(*x*), *E*(*x*), and *h*(*x*) are assumed to be constant functions of *x* in each vessel.

### Boundary Conditions

Because the heartbeat is approximately periodic, we assume that the blood pressure and flow rate are periodic in time, *i.e.*,

$$\begin{array}{c} p(x,0)=p(x,T),\;\;q(x,0)=q(x,T), \end{array}$$ (5)

where *T* is the period of one heartbeat and *T* = 0.75 *s* is used in this work.

**Fig 2:**
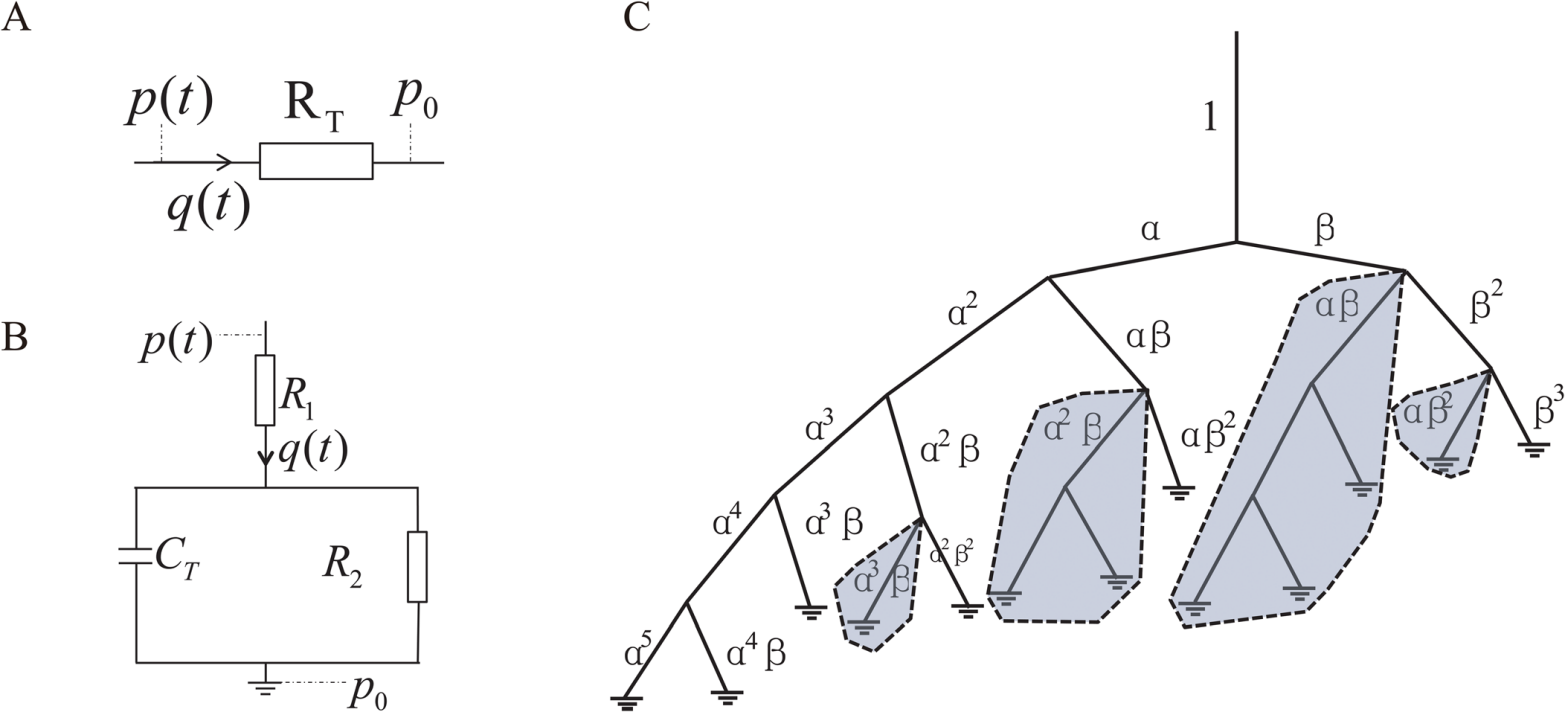
Illustrations of the outflow boundary conditions. A: the CR model. B: the three-element WK and CWK models. C: the ST model, where *α* and *β* are the the bifurcation ratios (see text).

**Fig 3:**
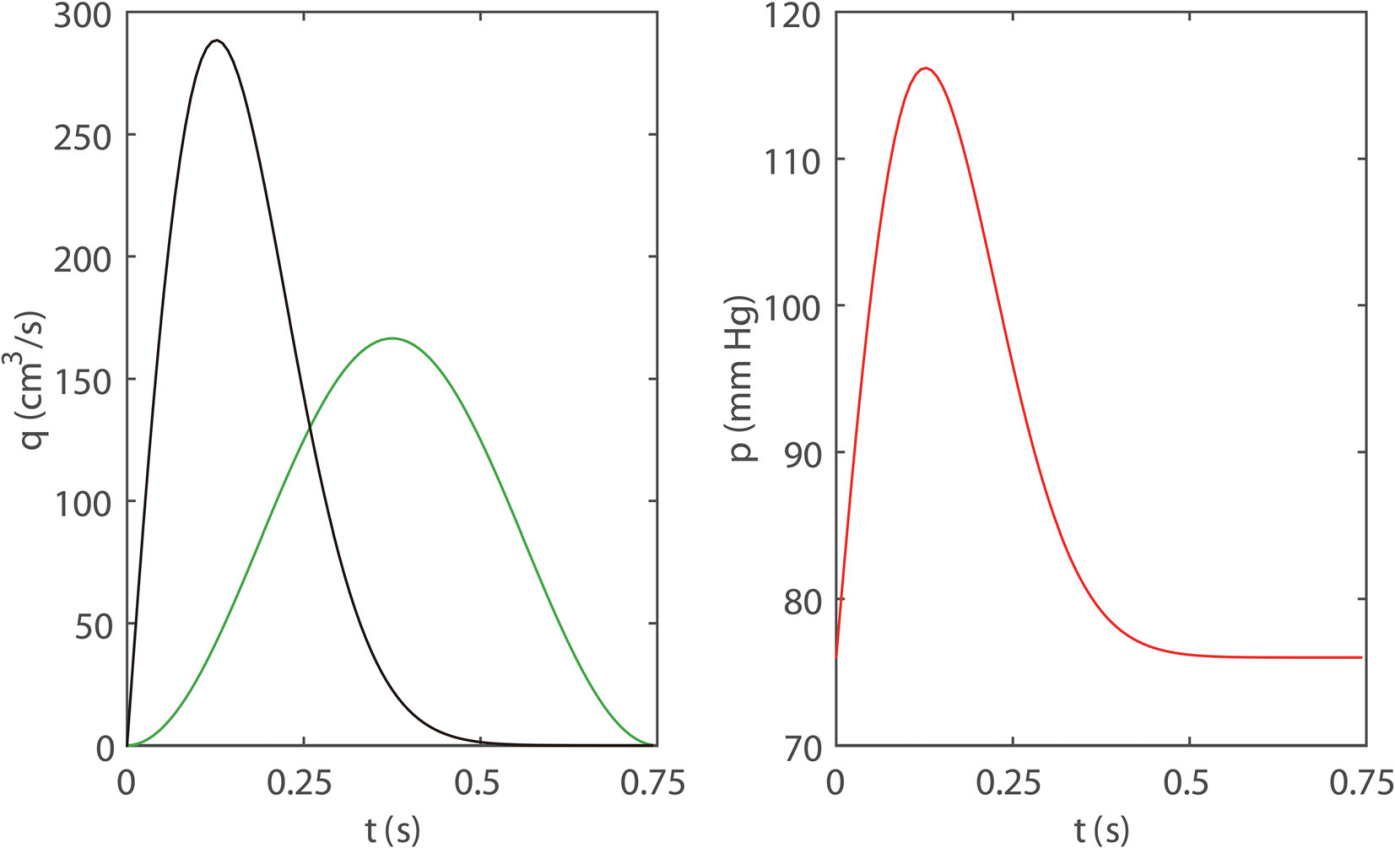
The three distinct inlet boundary conditions. The sinusoidal flow (Eq (27)), pulsatile flow (Eq (28)), and the pulsatile pressure (Eq (29)) inputs are represented by green, black, and red lines, respectively. The two flow inputs in the left panel are obtained with *Q*_0_ = 83.3 *cm* ^3^/*s*.

The spatial boundary conditions of a large arterial tree include one boundary condition at the inlet, one boundary condition at each outlet, and three boundary conditions at each bifurcation point. The specific outflow and inlet boundary conditions used in this work are shown in Figs 2 and 3, respectively.

**Boundary Condition at the Inlet** The cardiac output *Q*_I_(*t*), which can be measured experimentally, is used as the inlet boundary condition for the arterial tree [11, 17, 46],

$$\begin{array}{c} {uA|}_{x=0}=Q_{\text{I}}\left(t\right). \end{array}$$ (6)

**Bifurcation Boundary Condition** At a bifurcation point, the conservation of mass yields

$$\begin{array}{c} q_{P}\left(t\right)=q_{L}\left(t\right)+q_{R}\left(t\right), \end{array}$$ (7)

where the subscripts *P*, *L*, and *R* refer to the parent, the left-, and the right-daughter vessels, respectively. For blood flow, because the density of kinetic energy is much smaller than the pressure, the pressure can be regarded as being continuous at the bifurcation point [11],

$$\begin{array}{c} p_{P}\left(t\right)=p_{L}\left(t\right)=p_{R}\left(t\right). \end{array}$$ (8)

The conservation of mass and continuity of pressure can be naturally generalized to the case with more daughter vessels.

**Outflow Boundary Conditions** When the TR is sufficiently small, the nonlinearity of one-dimensional system (Eq (1)) in the truncated arterial crown is weak, because the ratio of the flow velocity to the pulse wave speed and the variation in the pulse wave speed are both very small [47]. Therefore, there is a linear relation between the blood pressure and flow rate at each outlet of the large arterial tree. In the Fourier space, the outflow boundary condition then satisfies

$$\begin{array}{c} \hat{P}\left(\omega \right)=\hat{Q}\left(\omega \right)Z\left(\omega \right), \end{array}$$ (9)

where $\hat{P}(\omega )$ is the Fourier mode of the blood pressure, *p*(*t*), and *Z*(*ω*) is the impedance of the truncated arterial crown. In the time domain, Eq (9) is equivalent to

$$\begin{array}{c} p\left(t\right)=\frac{1}{T}\int_{t-T}^{t}z\left(t-\tau \right)q\left(\tau \right)d\tau , \end{array}$$ (10)

where the kernel function, *z*(*t*), is the inverse Fourier transform of *Z*(*ω*). In the following, we describe three models of outflow boundary conditions—the constant resistance model, the Windkessel model, and the structured tree model.

*Constant Resistance Model*. In the CR model (Fig 2A), it is assumed that the impedance is constant for all frequencies [18–21, 48, 49],

$$\begin{array}{c} Z_{\text{CR}}\left(\omega \right)\equiv R_{T}\;\;\text{for}\;\text{any}\;\omega , \end{array}$$ (11)

where *R*_*T*_ is the total resistance. The corresponding kernel function is *z*_CR_(*t*) = *R*_*T*_ *Tδ*(*t*). Thus in the time domain, *p*(*t*) = *R*_*T*_ *q*(*t*). Under this boundary condition, there is no time lag between the blood pressure and flow rate at any outlet of the large arterial tree. This is inconsistent with experimental observations [5, 11].

*Three-element Windkessel Model*. A three-element WK model has been introduced to describe the relation between the blood flow and pressure at the outlet of the large arterial tree [6, 24, 38]. An analogy of the model to an electric circuit is illustrated in Fig 2B. In this analogy, the blood pressure, *p*(*t*), corresponds to the voltage in the electric circuit, and the blood flow rate, *q*(*t*), corresponds to the electric current. The capacitance, *C*_*T*_, describes the compliance of the downstream vasculature. By Kirchhoff’s law, from Fig 2B, it can be clearly seen that the blood pressure and flow rate in the three-element WK model satisfy

$$\begin{array}{c} \frac{dp(t)}{dt}+\frac{p}{R_{2}C_{T}}=R_{1}\frac{dq(t)}{dt}+\frac{q(R_{1}+R_{2})}{R_{2}C_{T}}, \end{array}$$ (12)

where *R*_1_ and *R*_1_ + *R*_2_ are the characteristic resistance and the total resistance, respectively [25, 37]. Therefore, at frequency *ω*, the impedance is

$$\begin{array}{c} Z_{\text{WK}}\left(\omega \right)=R_{1}+\frac{R_{2}}{1+i\omega C_{T}R_{2}}, \end{array}$$ (13)

and the corresponding kernel function is

$$\begin{array}{c} z_{\text{WK}}\left(t\right)=R_{1}T\delta \left(t\right)+\frac{T}{C_{T}[1-\exp(-\frac{T}{C_{T}R_{2}})]}\exp(-\frac{t}{C_{T}R_{2}}). \end{array}$$ (14)

*Structured Tree Model*. In the works of Refs. [17, 27, 28], a structured tree (Fig 2C) has been introduced to model a truncated arterial crown in order to obtain an outflow boundary condition at the outlet of the large arterial tree. In the structured tree, when the radius of a parent vessel, *R*_*P*_, is known, the radii of the left- and the right-daughter vessels are set to be *R*_*L*_ = *αR*_*P*_ and *R*_*R*_ = *βR*_*P*_, respectively, where *α* and *β* = (1 − *α* ^*ξ*^)^1/*ξ*^ are the bifurcation ratios, and *ξ* is the power in Murray’s law [11, 27, 28], $R_{p}^{\xi }=R_{L}^{\xi }+R_{R}^{\xi }$ (In the original Murray’s law, *ξ* is equal to 3). The length of a vessel in the structured tree is assumed to be proportional to its radius with length-to-radius ratio, *L*_r_. As in the work of Ref. [17], we use the structure with *α* = 0.9, *β* = 0.6, *ξ* = 2.7, and *L*_r_ = 50.0.

As mentioned above, when the radius of the root vessel is sufficiently small, the nonlinearity of Eq (1) in the structured tree is weak [47]. Therefore, the pulse wave can be described by the linearized system of Eq (1)

$$\begin{array}{cc} \rho \frac{\partial u}{\partial t}+\frac{\partial p}{\partial x} & =f,\\ C\frac{\partial p}{\partial t}+A_{0}\frac{\partial u}{\partial x} & =0, \end{array}$$ (15)

where

$$\begin{array}{c} C\approx {\frac{\partial A}{\partial p}|}_{A=A_{0}}=\frac{3A_{0}r_{0}}{2Eh} \end{array}$$ (16)

is the area compliance of the vessel wall. As discussed before, the boundary layer effect is important when the frequency is high in intermediate-sized vessels. The Womersley velocity profile [39, 50] can capture the boundary layer effect and is used to obtain the viscous term in Eq (15).

We use the following two recursive steps to obtain the impedance of a truncated arterial crown. Step one: with a known impedance *Z*(*L*, *ω*) at the outlet of a vessel, we are able to calculate the impedance at the inlet of the vessel [11, 17, 27, 28] through the Fourier transform of Eq (15)

$$\begin{array}{c} Z\left(0,\omega \right)=\frac{\lambda }{i\omega C}\frac{i\omega CZ(L,\omega )\cosh(\lambda L)+\lambda \sinh(\lambda L)}{\lambda \cosh(\lambda L)+i\omega C\sinh(\lambda L)Z(L,\omega )}, \end{array}$$ (17)

where $\lambda =\sqrt{\frac{-\rho C\omega^{2}}{A_{0}(1-F_{J})}}$. The real part of *λ* is the spatial decay rate of the magnitude of the wave and the imaginary part is the wave number. Step two: the total impedance of two parallel subtrees is

$$\begin{array}{c} Z_{P}\left(L,\omega \right)=\frac{Z_{L}\left(0,\omega \right)Z_{R}\left(0,\omega \right)}{Z_{L}\left(0,\omega \right)+Z_{R}\left(0,\omega \right)}, \end{array}$$ (18)

where *Z*_*P*_(*L*, *ω*), *Z*_*L*_(0, *ω*), and *Z*_*R*_(0, *ω*) are the impedances measured (or calculated) at the outlet of the parent vessel, the inlets of the left- and right-daughter vessels at the bifurcation point, respectively. By setting the impedance to be zero at the distal end of a structured arterial tree and using Eqs (17) and (18) recursively, the impedance, *Z*_ST_(*ω*), of the structured tree can be obtained in the end. Note that this recursive method can be used to calculate the impedance, *Z*_in_(*ω*), at the inlet of a truncated arterial crown once the geometrical structure and elastic properties of the truncated arterial crown are known.

### Parameter extraction and the complex Windkessel model

In the outflow boundary conditions for the blood flow in large arteries, the impedance reflects the geometrical and elastic details of the truncated arterial crown. From this point of view, the parameters in the simplified models, such as the three-element WK model, should be obtained from the impedance, *Z*_in_(*ω*), at the inlet of the truncated arterial crown. In this work, we are interested in a systematic procedure of parameter extraction from the impedance and an evaluation of the modeling error of pulse wave using the simplified models. In the following, we propose an improved three-element WK model with complex capacitance to reduce the modeling error of the pulse wave in large arteries.

From Eq (13), it can be seen that the total resistance, *R*_1_ + *R*_2_, is equal to *Z*_WK_(0), which is *Z*_in_(0), *i.e.*,

$$\begin{array}{c} R_{1}+R_{2}=Z_{\text{in}}\left(0\right). \end{array}$$ (19)

Furthermore, from Eq (13), the characteristic resistance, *R*_1_, satisfies *R*_1_ = lim_*ω* → ∞_ *Z*_WK_(*ω*) = lim_*ω* → ∞_ *Z*_in_(*ω*). It is known that

$$\begin{array}{c} R_{1}=\sqrt{\frac{\rho }{A_{\text{0r}}C_{\text{r}}}}=\frac{\rho c_{r}}{A_{\text{0r}}}, \end{array}$$ (20)

where *A*_0r_, *c*_*r*_, and *C*_r_ are the unstressed cross-sectional area, the pulse wave speed, and the area compliance of the root vessel, respectively [5, 38, 51]. In S1 Text, we provide an analytical derivation of Eq (20) with the Womersley velocity profile. Eq (20) shows that the characteristic resistance is determined by the compliant property of the root vessel and is independent of the structure of the downstream network. It implies that high-frequency wave transmission in the arterial network vanishes due to the boundary layer effect, which is consistent with the result in the work of Ref. [38]. This equation also provides a way to predict the compliance, in turn, the Young’s modulus through Eq (16), of the vessel wall by measuring the pulse wave speed of the vessel.

From Eqs (19) and (20), we can obtain the value of *R*_1_ and *R*_2_. For a three-element WK model, only the capacitance now remains to be determined. Note that in general only the low-frequency (for example, ∣*ω*∣ ≤ 10*ω*_0_, where *ω*_0_ is the fundamental frequency related to one heartbeat) components of the blood flow are significant (see S1 Fig). Therefore, the outflow boundary condition needs to capture the low-frequency impedances well. There have been various approaches to determine the capacitance [37, 52]. In the work of Ref. [52], the capacitance is derived from the constraint ∣*Z*_WK_(*ω*_0_)∣ = ∣*Z*_in_(*ω*_0_)∣ at the lowest nonzero frequency—the fundamental frequency, *ω*_0_, *i.e.*,

$$\begin{array}{c} C_{\text{T}}=\sqrt{\frac{{\left(R_{1}+R_{2}\right)}^{2}-{|Z_{\text{in}}\left(\omega_{0}\right)|}^{2}}{\omega_{0}^{2}R_{2}^{2}[{|Z_{\text{in}}\left(\omega_{0}\right)|}^{2}-R_{1}^{2}]}}. \end{array}$$ (21)

In the work of Ref. [37], to describe the impedance for the low-frequency response well, the capacitance is determined by the impedances of the two lowest nonzero frequencies, *ω*_0_ and 2*ω*_0_, *i.e.*,

$$\begin{array}{c} C_{\text{T}}=\sqrt{\frac{4{\left(R_{1}+R_{2}\right)}^{2}-{|Z_{\text{in}}\left(\omega_{0}\right)+Z_{\text{in}}\left(2\omega_{0}\right)|}^{2}}{9\omega_{0}^{2}R_{2}^{2}[{|Z_{\text{in}}\left(\omega_{0}\right)+Z_{\text{in}}\left(2\omega_{0}\right)|}^{2}-4R_{1}^{2}]}}. \end{array}$$ (22)

In our numerical test, the capacitances obtained with Eqs (21) and (22) are of no significant difference in the modeling error as studied below. Therefore, we will only use the one obtained using Eq (21) in the following.

To derive Eq (21), the magnitude of the impedance of the fundamental frequency in the WK model is assumed to be equal to ∣*Z*_in_(*ω*_0_)∣. However, this does not impose a constraint on the phase of the impedance, thus can also lead to a significant modeling error. To capture the phase of *Z*_in_(*ω*_0_) as well as its magnitude, we impose the constraint

$$\begin{array}{c} Z_{\text{CWK}}\left(\omega_{0}\right)=Z_{\text{in}}\left(\omega_{0}\right) \end{array}$$ (23)

instead, *i.e.*, $R_{1}+\frac{R_{2}}{1+i\omega_{0}R_{2}C_{T}}=Z_{\text{in}}(\omega_{0})$ (Eq (13)), to obtain the capacitance

$$\begin{array}{c} C_{\text{T}}=\frac{R_{1}+R_{2}-Z_{\text{in}}\left(\omega_{0}\right)}{iR_{2}\omega_{0}[Z_{\text{in}}\left(\omega_{0}\right)-R_{1}]}, \end{array}$$ (24)

where *C*_*T*_ can take on a complex value and is no longer a real number as in the standard WK model. The new model has the same physical parameters as the standard three-element WK model and will be referred to as the CWK model. Using this model, the phase of the wave of the fundamental-frequency becomes accurate. As will be shown below, this is important for reducing the modeling error. Since the kernel function is a real valued function by definition, the impedance of the CWK model, *Z*_CWK_(*ω*), is thus given by

$$\begin{array}{c} Z_{\text{CWK}}\left(\omega \right)=\{\begin{array}{cc} R_{1}+\frac{R_{2}}{1+iC_{\text{T}}R_{2}\omega }, & \text{for}\;\omega \geq 0,\\ \bar{Z_{\text{CWK}}\left(-\omega \right)}, & \text{otherwise,} \end{array} \end{array}$$ (25)

where $\bar{Z_{\text{CWK}}(\omega )}$ is the complex conjugate of *Z*_CWK_(*ω*). In addition, the parameter extraction method can be also invoked if the impedance is known from experiment, *e.g.*, through Eq (9).

### Modeling error

In general, it is difficult to obtain experimental measurement of the impedances. Below we use the impedances obtained from the ST model at the outlets as the reference to evaluate the error of the pulse wave using the WK and CWK models in the outflow boundary conditions. We define the modeling error

$$\begin{array}{c} \varepsilon_{M}\left(F\left(x\right)\right)=\frac{{∥F_{M}\left(x,\cdot \right)-F_{ST}\left(x,\cdot \right)∥}_{2}}{{∥F_{ST}\left(x,\cdot \right)∥}_{2}}\times 100\% \end{array}$$ (26)

for blood flow rate (*F* = *q*) and pressure (*F* = *p*) in the large arteries, where *M* = WK or CWK is used to represent results obtained through the WK or CWK model, and ‖*F*(*x*, ⋅)‖_2_ stands for the *L* ^2^ norm of *F* in one time period at position *x*.

## Results

To investigate the validity of the WK and CWK boundary conditions as approximations of truncated arterial crowns, we examine the modeling error in the blood pressure and flow rate in large arteries. The parameters of the WK and CWK models are obtained using Eqs (19)–(21) and (24). Since Eq (1) is a nonlinear system and there is no analytical periodic solution of Eq (1) in a large artery or an arterial tree, we use the numerical schemes and boundary condition treatments in the works of Refs. [11, 27] to obtain the solution of Eq (1). First, we discuss the blood pressure and flow rate in a single large artery with a truncated arterial crown (Fig 1A). Then, we consider those in the large systemic arterial tree (Fig 1B and 1C). Finally, we provide an estimate of the modeling error of pulse wave in large arteries using a characteristic time scale. In our simulation, three distinct cases as shown in Fig 3 are investigated, namely, two time-periodic inflow conditions and one time-periodic inlet-pressure boundary condition are imposed at the inlet of the single large artery or of the large arterial tree,

$$\begin{array}{ccc} Q_{c}\left(t\right) & = & Q_{0}[1-\cos(\omega_{0}t)], \end{array}$$ (27)

$$\begin{array}{ccc} Q_{p}\left(t\right) & = & 65.5Q_{0}t\left(T-t\right)\exp\left(-25t^{2}\right),\;\;\;\;\;\;\;\;\;\;\;\;\text{for}\;\;0\leq t\leq T, \end{array}$$ (28)

$$\begin{array}{ccc} P_{p}\left(t\right) & = & P_{0}\left(1.0+10.0t\left(T-t\right)\exp\left(-25t^{2}\right)\right),\;\;\text{for}\;\;0\leq t\leq T, \end{array}$$ (29)

where *Q*_0_ is the mean flow rate at the inlet of the single artery or the arterial tree and *P*_0_ = 76.0 *mmHg* is the diastolic blood pressure. The above three conditions will be referred to as the sinusoidal flow input, the pulsatile flow input, and the pulsatile pressure input, respectively (Fig 3).

### Case 1: Single artery

In the single artery case (Fig 1A), the wave propagation in the artery is obtained with the inflow boundary conditions given by Eqs (27)–(29) and the outflow boundary conditions obtained with the ST, WK, and CWK models, respectively. The typical values of the unstressed radius, *r*_0_, the length, *L*, of the vessel, and the mean flow rate, *Q*_0_, in the vessel are tabulated in Table 1.

**Table 1:** The geometrical data and the mean flow rate used in the single artery case.

*r*_0_ (*cm*)	0.35	0.30	0.26	0.20	0.16	0.13	0.12	0.11
*L* (*cm*)	17.5	15.0	13.0	10.0	8.0	6.5	6.0	5.5
*Q*_0_ (*cm* ^3^/*s*)	5.39	3.39	2.21	1.01	0.52	0.28	0.22	0.17

**Fig 4:**
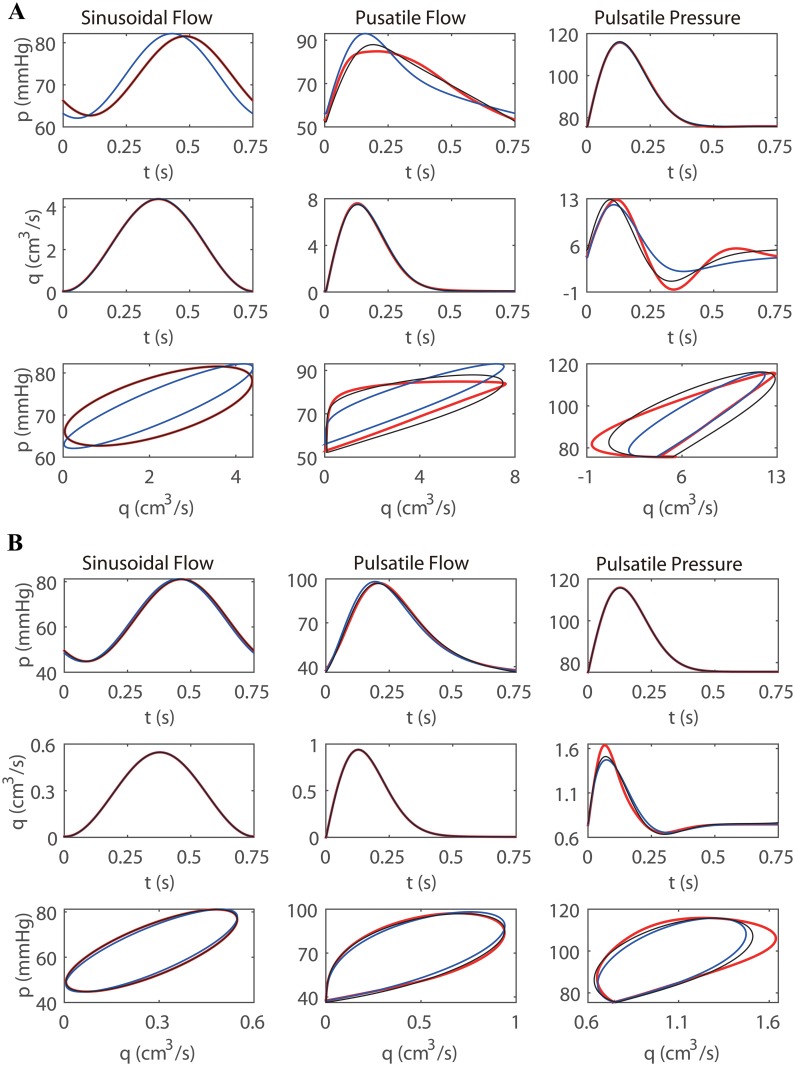
Time profiles and phase profiles of the blood pressure and flow rate at the mid-point of a single artery. The unstressed radii for Figure A and B are 0.26 *cm* and 0.13 *cm*, respectively. The left, middle, and right panels are obtained with the sinusoidal flow input (Eq (27)), the pulsatile flow input (Eq (28)), and the pulsatile pressure input (Eq (29)), respectively. The red, black, and blue lines correspond to the results obtained with ST, CWK, and WK models, respectively.

For the cases of *r*_0_ = 0.26 *cm* and *r*_0_ = 0.13 *cm*, the blood pressures and flow rates at the mid-point of each artery are shown in Fig 4. Comparing the results shown in Fig 4A and 4B, we can see that when the radius of the artery decreases from 0.26 *cm* (panels in Fig 4A) to 0.13 *cm* (panels in Fig 4B), both results obtained with the WK model and the CWK model result in a smaller modeling error. As is also shown in Fig 4, for the sinusoidal flow input (the left panels), the profiles obtained from the CWK model almost overlap with those obtained from the ST model. This is because that the fundamental-frequency impedances of the two models are identical (see Eq (23)). For the pulsatile flow input and the pulsatile pressure input (the middle and right panels in Fig 4), the CWK model also approximates the ST model significantly better than the WK model mainly because that the CWK model can accurately capture the fundamental-frequency time lag between the blood pressure and flow rate.

**Fig 5:**
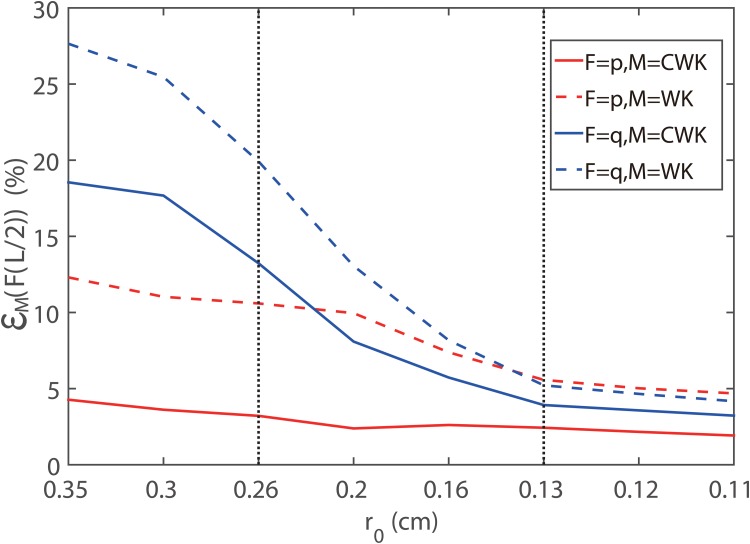
Modeling error induced by the WK and CWK models at the midpoint of a single artery. The *x*-axis is the unstressed radius of the single vessel. We compute the modeling errors of the blood pressure (red) and flow rate (blue) induced by the WK (dashed) and CWK (solid) model in the single artery case. The two vertical dotted lines indicate the radii used in Fig 4A and 4B, respectively.

In Fig 5, we collect the modeling errors of the blood pressure and flow rate at the mid-point of each single artery in Table 1, where the blood pressure is obtained with the pulsatile flow input and the blood flow rate is obtained with the pulsatile pressure input. The modeling errors of both blood pressure and flow rate decrease when the vessel radius decreases. The modeling error induced by the CWK boundary condition is smaller than that by the WK boundary condition. Note that under the requirement that the modeling error of the blood pressure be less than 5%, the radius of the vessel need to be smaller than 0.35 *cm* for the CWK model whereas smaller than 0.13 *cm* for the WK model.

### Case 2: Systemic arterial tree

The geometrical information of a truncated human large arterial tree (Fig 1B and 1C) is collected in Table 2 [17]. In our simulation, the pulsatile flow input with *Q*_0_ = 83.3 *cm* ^3^/*s* is imposed at the inlet of the arterial tree. In order to have an approximately uniform TR, a chosen radius, *R*_t_, is used to determine the modification of the original arterial tree in Table 2. If the radius of a terminal vessel of the original arterial tree is greater than *R*_t_, we attach a structured tree to the terminal vessel, thus obtaining a modified arterial tree. Then we delete all the vessels in the modified arterial tree whose parent-vessel radii are smaller than *R*_t_. This modified arterial tree with the ST model, the WK model, or the CWK model at all its outlets is used in our simulation to compute the modeling errors.

**Table 2:** Geometrical data of the human systemic large arterial system.

Vessel index	Artery name	*L*(*cm*)	*r*_0_(*cm*)	Vessel index	Artery name	*L*(*cm*)	*r*_0_(*cm*)
1	Ascending aorta	3.00	1.440	26	L. Posterior tibial	32.00	0.247
2	Aortic arch	2.00	1.353	27	L. Anterior tibial	34.25	0.130
3	L. Common carotid	20.75	0.370	28	R. External iliac	5.75	0.368
4	Aortic arch	4.00	1.300	29	R. Femoral	14.50	0.347
5	L. Subclavian	3.50	0.423	30	R. Femoral	44.25	0.299
6	L. Brachial	42.25	0.403	31	R. Posterior tibial	32.00	0.247
7	L. Ulnar	6.75	0.215	32	R. Anterior tibial	34.25	0.130
8	L. Ulnar	17.00	0.203	33	R. Deep femoral	12.50	0.255
9	L. Interosseous	8.00	0.091	34	R. Internal iliac	5.00	0.200
10	L. Radial	23.50	0.174	35	Inferior mesenteric	5.00	0.160
11	L. Vertebral	14.75	0.188	36	R. Renal	3.25	0.260
12	Thoracic aorta	5.25	1.194	37	Celiac axis	1.00	0.390
13	Thoracic aorta	10.50	1.071	38	Hepatic	6.50	0.220
14	Abdominal aorta	5.25	0.861	39	Hepatic	1.00	0.220
15	Superior mesenteric	6.00	0.435	40	Intercostals	8.00	0.200
16	Abdominal aorta	1.00	0.772	41	Brachiocephalic	3.50	0.620
17	Abdominal aorta	1.00	0.756	42	R. Common carotid	17.75	0.370
18	L. Renal	3.25	0.260	43	R. Subclavian	3.50	0.423
19	Abdominal aorta	10.00	0.740	44	R. Vertebral	14.75	0.188
20	Abdominal aorta	7.00	0.601	45	R. Brachial	42.25	0.403
21	L. External iliac	5.75	0.368	46	R. Radial	23.50	0.174
22	L. Internal iliac	5.00	0.200	47	R. Ulnar	6.75	0.215
23	L. Femoral	14.50	0.347	48	R. Interosseous	8.00	0.091
24	L. Deep femoral	12.50	0.255	49	R. Ulnar	17.00	0.203
25	L. Femoral	44.25	0.299				

The Vessel index is used in Fig 1B and 1C to label the vessel. *L* and *r*_0_ are the fixed length and unstressed radius of each vessel, respectively. The data are adapted from the geometrical data in Ref. [17]

**Fig 6:**
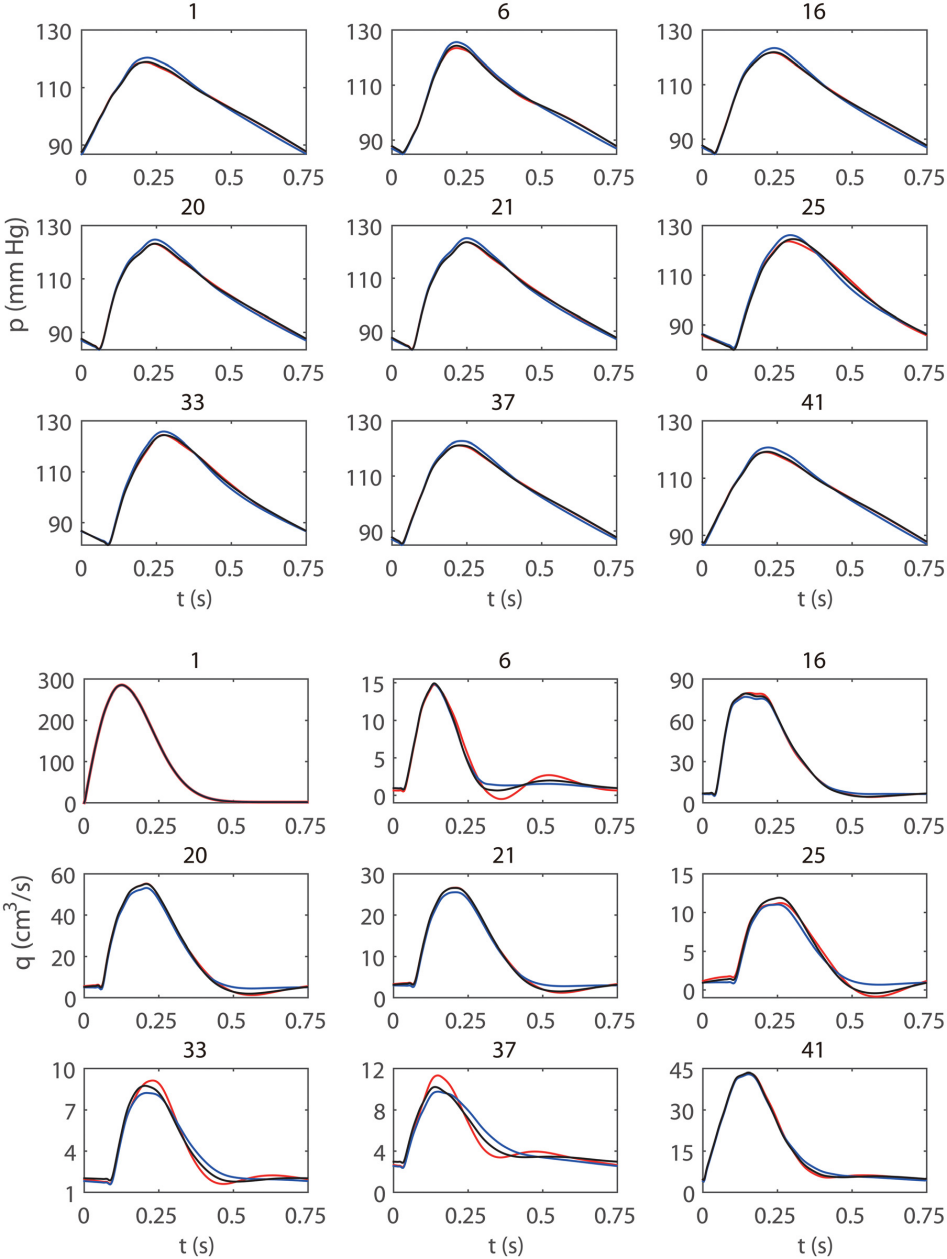
The blood pressures and flow rates in the systemic arterial tree. The line conventions are the same as those in Fig 4. The blood pressure and flow rate are measured at the midpoint of each of 9 representative vessels, whose corresponding vessel indices as listed in Table 2 are labeled on the top of each panel.

In Fig 6, we display the blood pressures and flow rates at the mid-point of each of 9 representative vessels in the large arterial tree obtained with *R*_t_ = 0.25 *cm*. From Fig 6, it can be seen that the profiles of the blood pressure and flow rate obtained from the WK model and from the CWK model are in good agreement with those obtained with the ST model. The CWK model again gives rise to a smaller modeling error than the WK model in the modeling of the large arterial tree.

**Fig 7:**
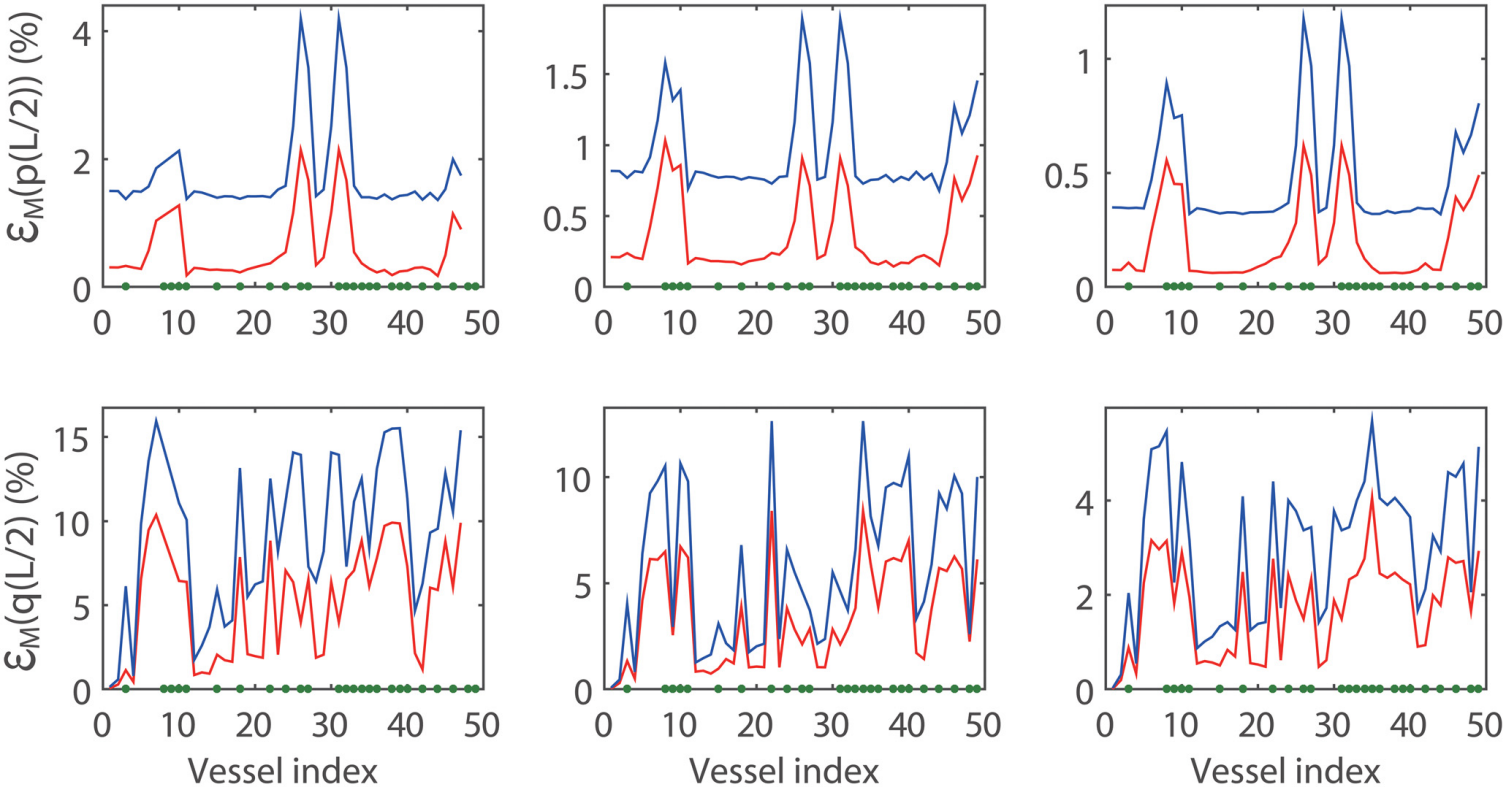
Modeling error induced by the CWK model and the WK model in three systemic arterial trees. The red and blue lines represent the modeling errors induced by the CWK model and the WK model, respectively. The *x*-axis is the vessel index in Table 2. There is a total of 83, 149, and 335 vessels in the three arterial trees with *R*_*t*_ = 0.25 *cm* (left panel), *R*_*t*_ = 0.20 *cm* (middle panel), and *R*_*t*_ = 0.15 *cm* (right panel), respectively. The terminal vessels of the original arterial tree shown in Fig 1B and 1C are labeled by the green dots on the *x*-axis.

To clarify the modeling errors induced by the WK and CWK boundary conditions, we collect the modeling errors of the blood pressure and flow rate at the mid-point of each vessel in the truncated arterial tree in Fig 7 and list the average errors in Table 3. As expected, it can be seen that when the threshold, *R*_t_, decreases, the modeling errors of both the blood pressure and flow rate decrease. The modeling error of the flow rate is much greater than that of the blood pressure. This difference in modeling error between the blood pressure and flow rate is partly due to the fact that ratio of the maximal fluctuation to the mean value of the blood pressure is much smaller than that of the blood flow rate. As can be seen from Fig 7, the modeling errors of the blood pressure and flow rate are large in certain terminal vessels of the arterial tree. On the other hand, the modeling errors of the blood pressure and flow rate are small in the upstream vessels (according to the direction of the blood flow). In fact, this can be understood by the analysis of the error propagation (see S2 Text)—the modeling error is compressed when it propagates upstream. Therefore, the modeling errors of blood pressure and flow rate in a large arterial system can be bounded by those at the outlets of the large arterial tree.

**Table 3:** Average modeling errors of blood pressure and flow rate induced by the CWK and WK models in large arterial trees.

*R*_*t*_ (*cm*)	Error in *p* by CWK(%)	Error in *p* by WK(%)	Error in *q* by CWK(%)	Error in *q* by WK(%)
0.25	0.57	1.75	5.01	9.11
0.20	0.37	0.96	3.48	5.71
0.15	0.20	0.48	1.74	2.99

### Modeling error evaluation

**Fig 8:**
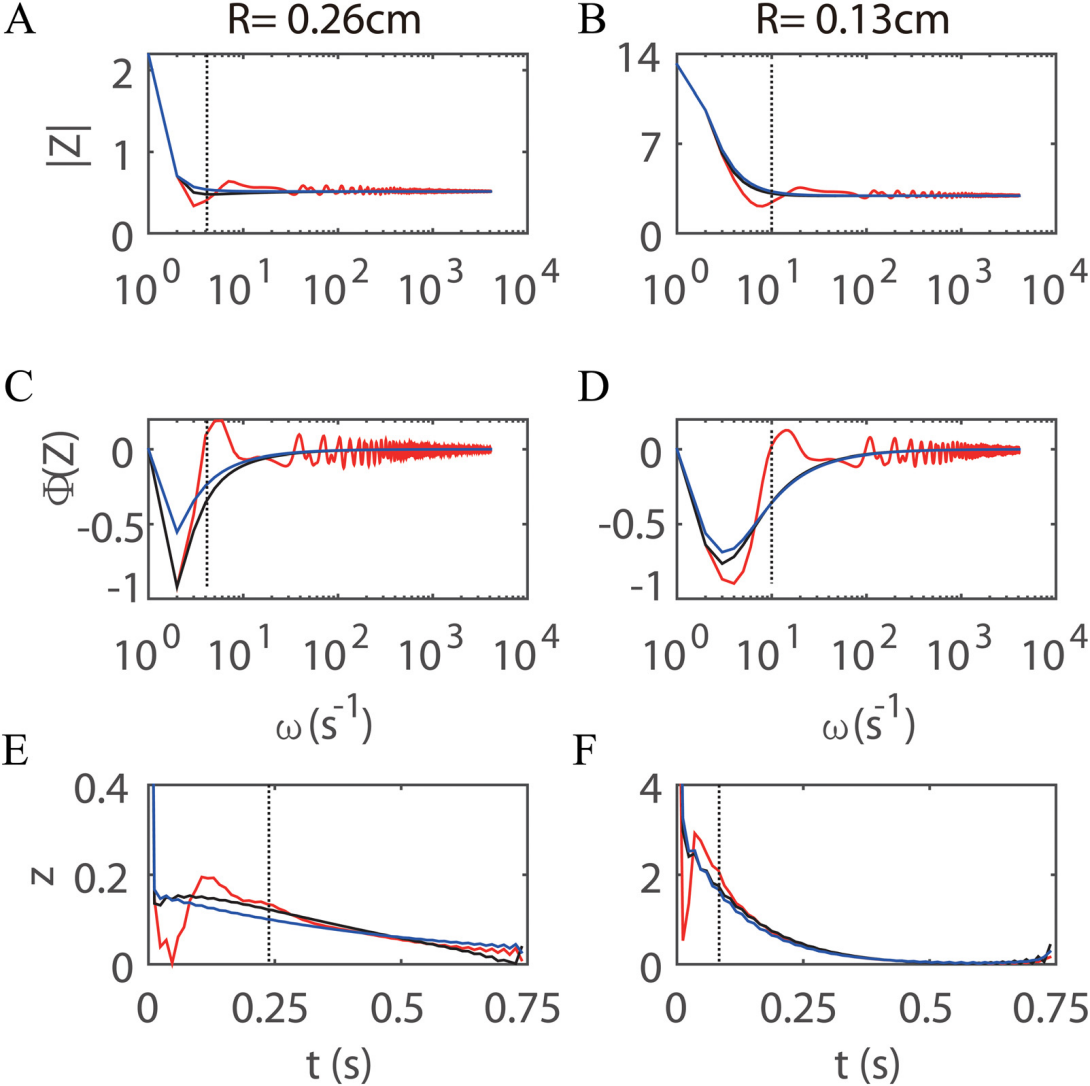
The impedance and kernel function obtained from the ST model and from the corresponding WK and CWK models. The line conventions are the same as those in Fig 4. The root-vessel radii are *R* = 0.26 *cm* and *R* = 0.13 *cm* for the left and the right panels, respectively, as marked on the top of the panels. The units of magnitude (A and B), phase (C and D) of the impedance, and the kernel function (E and F) are 10^4^ *gcm* ^−4^ *s* ^−1^, *rad*, and 10^5^ *gcm* ^−4^ *s* ^−1^, respectively. The vertical dotted line refers to the characteristic frequency in A-D and the corresponding characteristic time in E and F.

To gain an intuitive understanding of the modeling error of the wave propagation in large arteries induced by the outflow boundary conditions, we compare the impedance and the kernel function obtained from the ST model with those obtained from the corresponding WK and CWK models, whose parameters are extracted from the impedance obtained from the ST model using Eqs (19)–(21) and (24). The magnitude, phase of the impedance, and the kernel function are shown in Fig 8.

As can be seen from Fig 8A–8D, for both very low frequencies and very high frequencies, profiles of the impedances of the three models are in good agreement with one another. However, for intermediate frequencies (around the location of the minimal magnitude of the impedance of the ST model in Fig 8A and 8B), there is a large discrepancy. When the root-vessel radius (TR of the large arterial tree) of the structured tree decreases from 0.26 *cm* (Fig 8A and 8C) to 0.13 *cm* (Fig 8B and 8D), there is no significant decrease in the largest discrepancy of impedances. Instead, the location of the minimal impedance has shifted to a higher frequency. As a result of this frequency shift, the low-frequency components of impedance are captured more accurately in vessel trees with smaller TR. As discussed before, these low-frequency components are important for describing the wave reflection effect.

In order to understand the frequency shift, we turn to the discussion of a characteristic time scale, *τ*_c_, of a truncated arterial crown, which is defined as the time for a pulse wave to propagate from the inlet of the truncated arterial crown to its distal ends and then reflected back to the inlet. The characteristic time scale and the corresponding characteristic frequency, *ω*_c_, for a truncated arterial crown can be estimated by

$$\begin{array}{c} \tau_{\text{c}}=\frac{2L_{\text{e}}}{c_{\text{e}}},\;\;\text{and}\;\;\omega_{\text{c}}=\frac{2\pi }{\tau_{\text{c}}}, \end{array}$$ (30)

where *L*_e_ is the length of the longest branch of the arterial crown and *c*_e_ is the effective pulse wave speed of the arterial crown. Under the physiological condition, the pulse wave speed is not very sensitive to vessel radius and we can use the pulse wave speed of the root vessel to estimate the characteristic time scale.

The characteristic frequency and time of the structured tree are marked by the dotted line in Fig 8A, 8B, 8E and 8F, respectively. As can be seen from Fig 8A–8D, the large discrepancy of the impedances between different models is concentrated around the characteristic frequency. The large discrepancy shifts with the characteristic frequency when the root-vessel radius of the arterial crown decreases.

**Fig 9:**
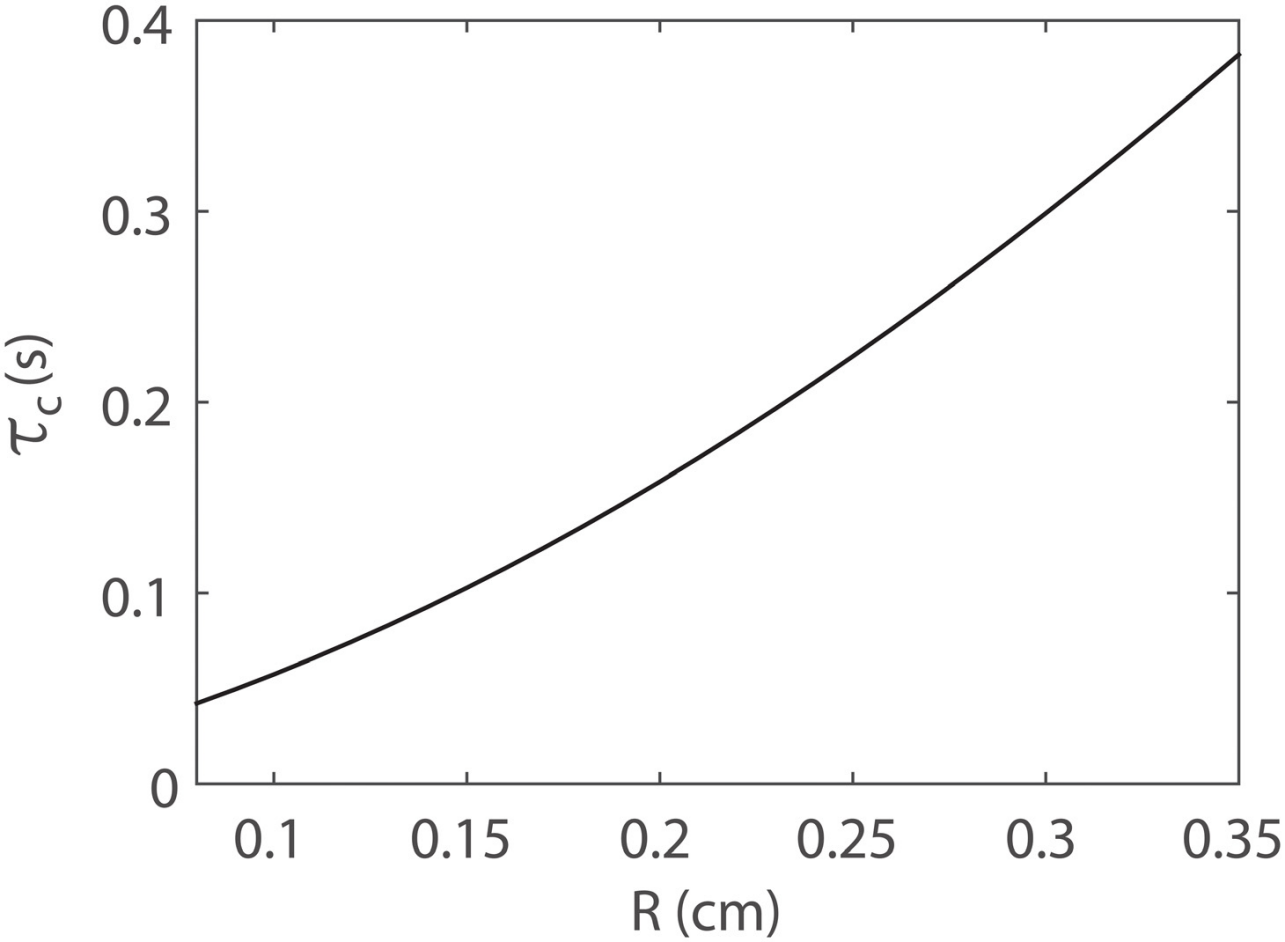
The characteristic time, *τ*_*c*_, of structured trees as a function of root vessel radius *R*. In the estimate of *τ*_*c*_ using Eq (30), the length of the longest branch *L* is 10 times the length of the root vessel and *c*_e_ is chosen to be the pulse wave speed of the root vessel.

From Fig 8E and 8F, we can see that the error induced by the WK and CWK models in the kernel function lies mainly in the interval (0, *τ*_c_). According to the Fourier transform, the mean value of the kernel function is equal to the total resistance. Because the total resistances of the WK and CWK models are accurate by construction (Eq (19)), the mean value of the kernel functions of the ST, WK, and CWK models are identical. As a result, the error induced by the WK and CWK models by using Eq (10) is approximately proportional to $\tau_{\text{c}}^{2}$. As shown in Fig 9, when the root-vessel radius of the arterial crown decreases, the characteristic time scale also decreases. Therefore, the modeling error of the pulse wave decreases when the root vessel radius decreases. This is consistent with our numerical results.

## Discussion

There have been many works dealing with parameter extraction for the WK model, including the low-frequency impedance method [37, 52], the integral method [53], and the estimation of capacitance based on an inverse proportionality between the peripheral total resistance and the capacitance [38]. For the parameter of the total resistance, our result is the same as that in the previous works of Refs. [37, 38, 53]. For the characteristic resistance, *R*_1_ is averaged over a number of high-frequency impedances at the outlet of the large artery in Refs. [37, 52, 53]. In our work, we have shown that the characteristic resistance can be estimated with the pulse wave speed or the area compliance through Eq (20). Note that the pulse wave speed can be measured by wave intensity analysis [54, 55]. In general, there are errors in experimentally measured high-frequency blood pressure and flow rate and they may cause a large error in the high-frequency impedance estimated by $Z(\omega )=\frac{\hat{P}(\omega )}{\hat{Q}(\omega )}$. Our approach can avoid the error caused by the experimental measurement of high-frequency blood pressure and flow rate. Furthermore, we have demonstrated that the complex capacitance in our CWK model can be used to capture the phase as well as the magnitude of the impedance of the fundamental frequency accurately. As a consequence, the complex capacitance can help to reduce the modeling error of the pulse wave. However, the parameter extraction of the WK and CWK model considers only the fundamental-frequency impedance to estimate the capacitance. It is necessary to take into account more low-frequency impedances to further reduce the modeling error.

When the root-vessel radius of the arterial crown decreases, the effective pulse wave speed, *c*_*e*_, increases and the length, *L*_*e*_, of the longest branch of the arterial crown decreases. As a result, the characteristic time scale, *τ*_*c*_, decreases. Thus the modeling error of the pulse wave in large arteries induced by WK and CWK models decreases when the root-vessel radius of the arterial crown decreases. Using the modeling error of the pulse wave induced by the WK model in the single artery case, we can roughly estimate that the modeling error in the work of Ref. [6] is smaller than 10.0% for the blood pressure and 19.92% for the blood flow rate, based on the fact that the largest terminal vessel radius of the arterial tree used there is 0.26 *cm*.

For comparison, we have also used a random tree to evaluate the modeling errors of blood pressure and flow rate in large arteries. The results are included in S3 Text. The modeling errors of blood pressure and flow induced by the WK and CWK models with the reference impedance obtained from the random tree are on the same order as those obtained from the ST model. However, so far, all the comparisons are based on the impedance obtained from tree models. It is necessary to compare with *in vivo* data to further validate our results in the future.

## Conclusion

In our work, we have discussed a systematic methodology to extract parameters of the three-element WK model from the impedance of a truncated arterial crown or from experimental measurements of the blood pressure and flow rate at the outlet of a large arterial tree. To capture the fundamental-frequency time lag between the blood pressure and flow rate, a complex capacitance is introduced in our CWK model. From our numerical results and error evaluation, we have demonstrated that a smaller truncation radius leads to a smaller modeling error and that the modeling error induced by the CWK model is significantly smaller than that by the WK model for the same TR. As a result, the CWK model allows for a greater truncation radius than the WK model for a similarly required modeling accuracy, thus can reduce the task of experimental measurement of the vessel geometry.

## Supporting Information

S1 Text The analytical derivation of the characteristic resistance, *R*_1_. (PDF)

S2 Text Propagation of the modeling error in an arterial tree. (PDF)

S3 Text The comparison of blood flow in single vessels between the random tree model and the ST, WK, or CWK models. (PDF)

S1 Fig The blood flow rate measured in the ascending aorta experimentally. *ω*_*k*_ in the x-axis of the right panel is *ω*_*k*_ = *kω*_0_. The blood flow rate is measured by an automated contour tracing method in the work of Ref. [56]
(PDF)
