# Supplementary material for: Outflow Boundary Conditions for Blood Flow in Arterial Trees
Source: PLoS One. 2015 May 22;10(5):e0128597. doi: 10.1371/journal.pone.0128597 (PMC4441455; doi:10.1371/journal.pone.0128597)
Supplement: S1 Text — (PDF) [file pone.0128597.s001.pdf]

The analytical derivation of the characteristic resistance, R_1

At the inlet of the root vessel of a truncated arterial crown, the impedance with the Womersley velocity profile is

$$Z(0,\omega) = \frac{\lambda}{i\omega C}\frac{i\omega C Z(L,\omega)\cosh(\lambda L) + \lambda\sinh(\lambda L)}{\lambda\cosh(\lambda L) + i\omega C\sinh(\lambda L)Z(L,\omega)}, \tag{1}$$

where $Z(L,\omega)$ is a known impedance at the outlet of the root vessel and $\lambda = \sqrt{\frac{-\rho C\omega^2}{A_0(1-F_J)}}$ is the wave number as shown in the main text. According to the relation between the trigonometric functions and the hyperbolic functions, equation (1) can be rewritten as

$$Z\left(0,\omega\right) = \frac{\lambda}{\omega C}\frac{\dfrac{\omega C}{\lambda}Z\left(L,\omega\right) - \tan\left(i\lambda L\right)}{1 + \dfrac{\omega C}{\lambda}Z\left(L,\omega\right)\tan\left(i\lambda L\right)} = \frac{\lambda}{\omega C}\tan\left(\phi - i\lambda L\right), \tag{2}$$

where ϕ is defined by $\tan\phi = \frac{\omega C Z(L,\omega)}{\lambda}$. Let $a = \mathrm{Re}(\phi - i\lambda L)$, $b = \mathrm{Im}(\phi - i\lambda L)$, then we have

$$Z\left(0,\omega\right) = \frac{\lambda}{\omega C}\tan\left(a+ib\right) = \frac{\lambda}{\omega C}\frac{\tan a + i\tanh b}{1 - i\tan a\tanh b}. \tag{3}$$

When the frequency ω is large, the Womersley number, $W = r_0\sqrt{\omega\rho/\mu}$, is also large, therefore, we have the following approximation:

$$F_J(W) \sim \frac{2}{Wi^{0.5}}\left(1 + \frac{1}{2W}\right),\quad \lambda \sim \sqrt{\frac{\rho C}{A_0}}\left(-i\omega + \sqrt{\frac{\omega\gamma}{\rho}}\frac{1+i}{4}\right), \tag{4}$$

where $\gamma = \frac{8\pi\mu}{A_0}$. Then we have $\mathrm{Im}(i\lambda L) \to \infty$ and $|b| \to \infty$ as $\omega \to \infty$. Thus, $\tanh b \to \frac{|b|}{b}$ and $\frac{\tan(a)+i\tanh(b)}{1-i\tan(a)\tanh(b)} \to i$ as $\omega \to \infty$. Finally, we can obtain

$$R_1 = \lim_{\omega\to\infty} Z\left(0,\omega\right) = \sqrt{\frac{\rho}{A_0 C}}. \tag{5}$$

With the expression of the pulse wave speed, $c = \sqrt{\dfrac{2Eh}{3\rho r_0(x)}}$, and the area compliance, $C = \frac{3A_0 r_0}{2Eh}$, in the main text, we can also arrive at

$$R_1 = \frac{\rho c}{A_0}. \tag{6}$$
