# Supplementary material for: Outflow Boundary Conditions for Blood Flow in Arterial Trees
Source: PLoS One. 2015 May 22;10(5):e0128597. doi: 10.1371/journal.pone.0128597 (PMC4441455; doi:10.1371/journal.pone.0128597)
Supplement: S2 Text — (PDF) [file pone.0128597.s002.pdf]

Propagation of the modeling error in an arterial tree

In this part, we consider the error propagation in the large arteries due to the error of impedance in the outflow boundary condition. Since the nonlinearity is weak as mentioned in the main text, we will use the linearized system to consider the error propagation. For a given vessel, the modeling error is a linear combination of the error of the impedance at the outlet and the error of the pressure at the inlet. Therefore, we only consider the propagation of the modeling error of impedance and blood pressure. Our analysis shows that the modeling error can be bounded by the errors of the impedances at the outlets.

Error of Impedance

In order to estimate the error of the impedance in the large arterial tree, we need to find: 1. the error of the impedance at the inlet of a vessel with given error of the impedance at the outlet of the vessel; 2. the error in the total impedance of two parallel vessel crowns with given errors in the impedances of the two vessel crowns.

First, if we have $\widetilde{Z}(L,\omega) = Z(L,\omega)(1+\varepsilon)$ at the outlet, where ε is the relative error of the impedance at the outlet, we assume that the impedance at the inlet of the vessel satisfies $\widetilde{Z}(0,\omega) = Z(0,\omega)\left(1+\phi_1(\omega)\varepsilon + \mathrm{O}(\varepsilon^2)\right)$. According to the impedance in a single vessel in the main text, we have

$$\begin{aligned}\phi_1(\omega) &= \frac{\partial \ln(Z(0,\omega))}{\partial Z(L,\omega)} \times Z(L,\omega) \\ &= \frac{1}{1+\frac{\lambda}{i\omega C Z(L,\omega)}\tanh(\lambda L)} - \frac{1}{1+\frac{\lambda}{i\omega C Z(L,\omega)}\coth(\lambda L)}.\end{aligned} \tag{1}$$

In Eq. (1), the magnitude of $\phi_1(\omega)$ depends on the radius of the vessel and the impedance at the outlet. The magnitudes of $\phi_1(\omega_0)$ and $\phi_1(8\omega_0)$ for different vessel radius are shown in Figs. 1 and 2, respectively, where ω_0 is the fundamental frequency. As can be seen from the two figures, the error at the inlet is compressed ($|\phi_1(\omega)| < 1$) in most cases under the physiological condition.

Second, if the relative error of the impedances of the two parallel vessel crowns, $Z_\mathrm{l}(\omega)$ and $Z_\mathrm{r}(\omega)$, are ε_1 and ε_2, respectively, then the relative error of the total impedances of the two vessel crowns is $\frac{Z_l\varepsilon_2+Z_r\varepsilon_1}{Z_l+Z_r}$. Generally, there is no significant magnification of the error in the total impedance comparing to the errors of the impedances of the two vessel crowns.

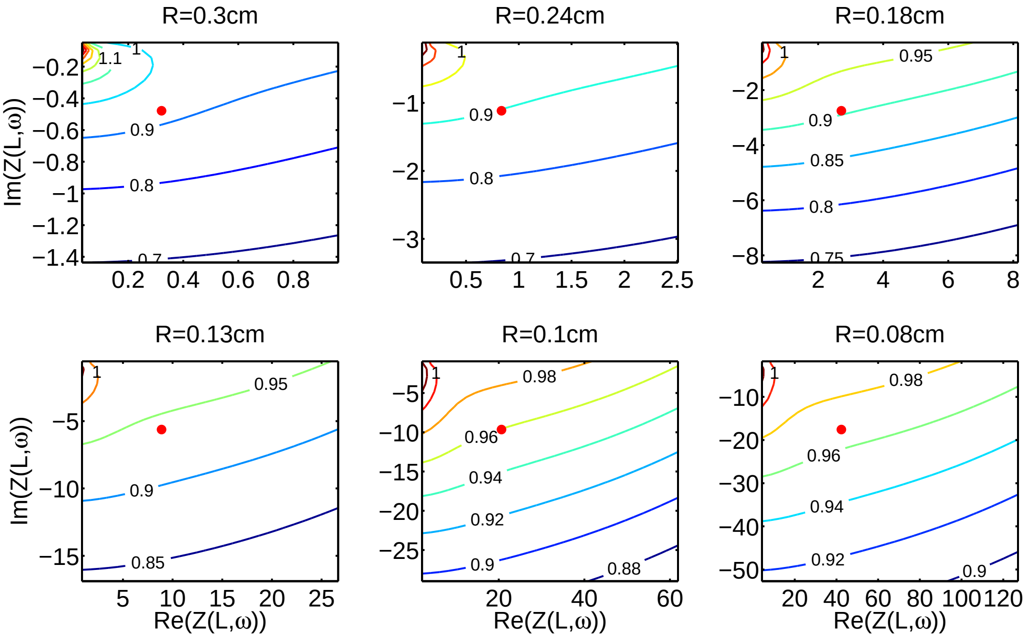

Figure 1. The contour plot of the magnitude $|\phi_1(\omega_0)|$ for different vessel radii and impedances at the outlet. The x- and y-axis are the real part and imaginary part of $Z(\omega, L)$, respectively. The unit of the impedances is $10^4\ gcm^{-4}s^{-1}$. The red dot indicates the error for the case that the impedance $Z(\omega, L)$ is obtained with the structured tree.

Error of pressure

Next, we consider the error of the blood pressure at the end of a vessel with a given error of the blood pressure at the inlet and a given error of the impedance at the outlet. The linearized system of the blood pressure and the blood flow rate (in the Fourier domain) in a vessel satisfies

$$\begin{cases} \zeta\widehat{Q} + \widehat{P}_x = 0, \\ i\omega C\widehat{P} + \widehat{Q}_x = 0, \end{cases} \tag{2}$$

where $\zeta = \frac{i\rho\omega}{A_0(1-F_J)}$. With the boundary conditions $\widehat{P}(0,\omega) = P(\omega)$ and $\widehat{P}(L,\omega) = Z(L,\omega)\widehat{Q}(L,\omega)$ in the vessel, we have

$$\widehat{P}(L,\omega) = \frac{\lambda}{\zeta}\frac{Z(L,\omega)P(\omega)}{\sinh(\lambda L) + \frac{\lambda}{\zeta}\cosh(\lambda L)Z(L,\omega)}. \tag{3}$$

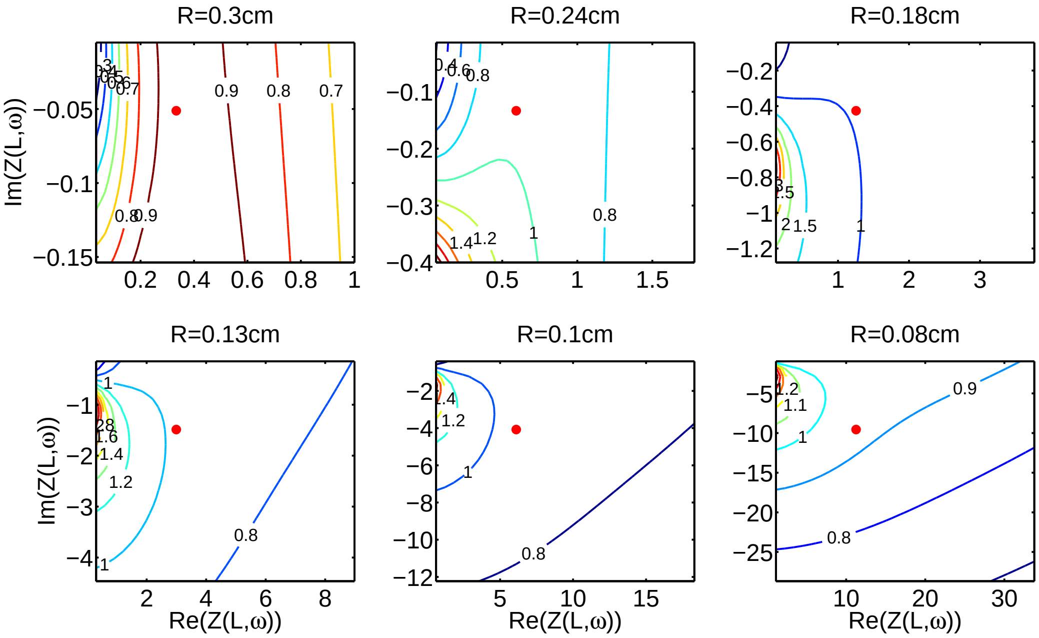

Figure 2. The contour plot of the magnitude $|\phi_1(8\omega_0)|$ for different vessel radii and impedances at the outlet. The axis conventions are the same as those in Fig. 1.

Assuming $\widetilde{P}(\omega) = P(\omega)(1+\varepsilon_1)$ and $\widetilde{Z}(L,\omega) = Z(L,\omega)(1+\varepsilon_2)$, then we have

$$\widetilde{P}(L,\omega) = \widehat{P}(L,\omega)\left(1+\varepsilon_1+\phi_2(\omega)\varepsilon_2\right), \tag{4}$$

where

$$\phi_2(\omega) = \frac{\sinh(\lambda L)}{\sinh(\lambda L) + \dfrac{\lambda}{\zeta}\cosh(\lambda L)\, Z(L,\omega)}. \tag{5}$$

Therefore, the error of the pressure at the outlet includes two parts: the first part inherits directly from the error of the pressure at the inlet of the vessel and the second part comes from the error in the impedance at the outlet.

In Figs. 3 and 4, we show the magnitude of $\phi_2(\omega)$ for different radii caused by the error of the impedance at the outlet. For the fundamental frequency, $|\phi_2(\omega_0)|$ is very small. For the intermediate frequencies, $|\phi_2(\omega)|$ can be relatively large but smaller than 1 for most cases. Thus the modeling error of the blood pressure can be bounded by the error of the impedance at the outlet.

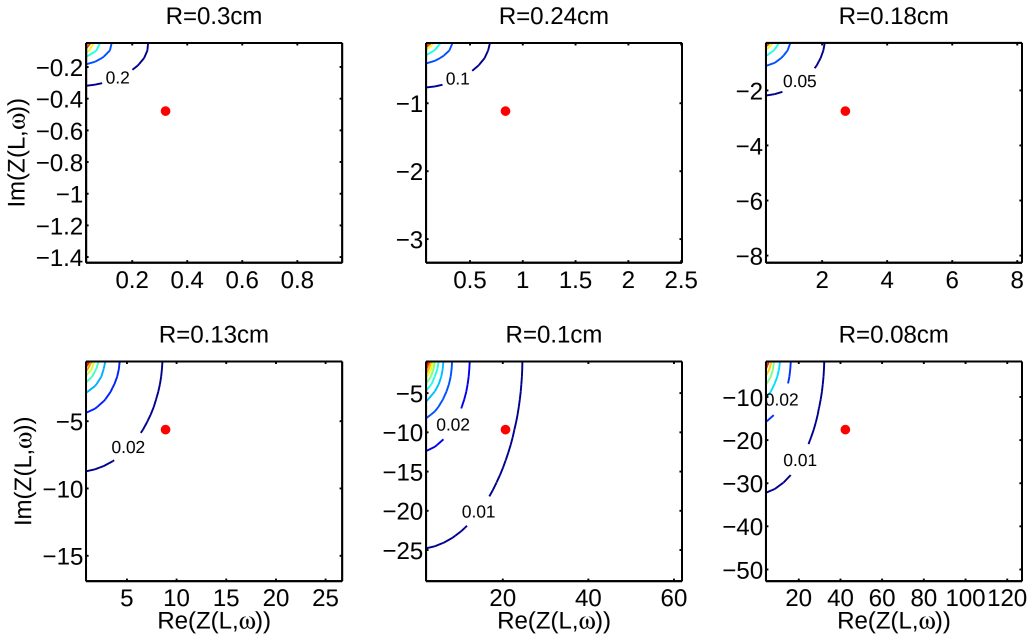

Figure 3. The contour plot of the magnitude $|\phi_2(\omega_0)|$ for different vessel radii and impedances at the outlet. The axis conventions are the same as those in Fig. 1.

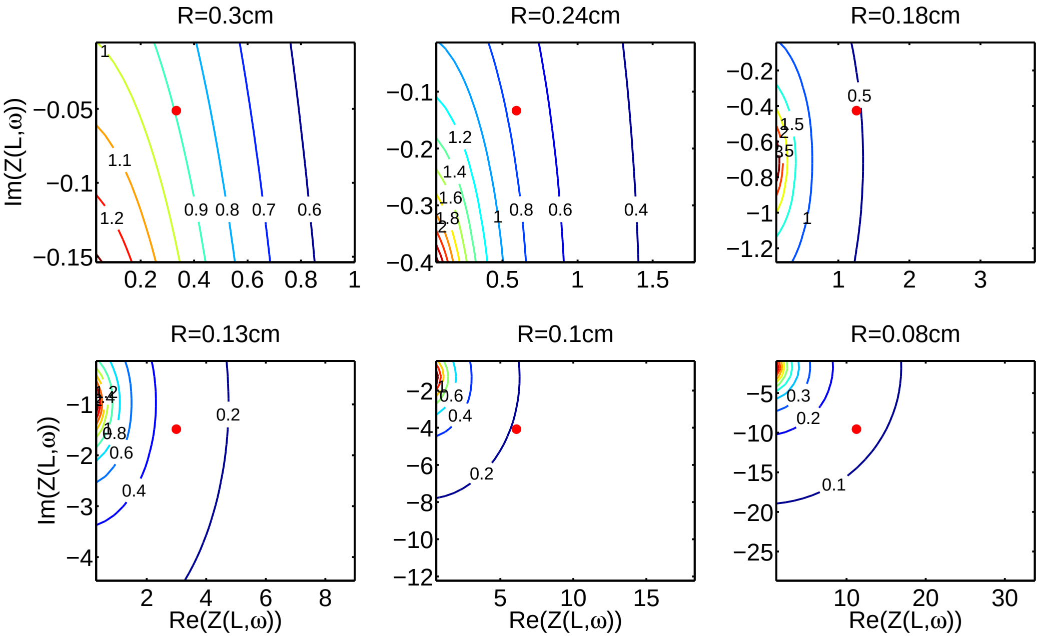

Figure 4. The contour plot of the magnitude $|\phi_2(8\omega_0)|$ for different vessel radii and impedances at the outlet. The axis conventions are the same as those in Fig 1.
