# Supplementary material for: Outflow Boundary Conditions for Blood Flow in Arterial Trees
Source: PLoS One. 2015 May 22;10(5):e0128597. doi: 10.1371/journal.pone.0128597 (PMC4441455; doi:10.1371/journal.pone.0128597)
Supplement: S3 Text — (PDF) [file pone.0128597.s003.pdf]

The comparison of blood flow in single vessels between the random tree model and the ST, WK, or CWK models

In the main text, we have considered the modeling error of the pulse wave in large arteries induced by the WK and CWK models when the impedance of the ST model is used as the reference. It is also of interest to examine whether other structural properties of the arterial tree are also significant, such as the bifurcation ratio of the vessel tree. In the following, we study the impedance of a random tree with random bifurcation ratios, where the random tree is constructed by the following rules:

1. The vessel tree is a binary tree;

2. The radii of a parent vessel and its two daughter vessels, R_{l}, R_{r}, R_{p}, satisfy $R_{\mathrm{l}}^{\xi} + R_{\mathrm{r}}^{\xi} = R_{\mathrm{p}}^{\xi}$, where $\xi = 2.7$ is used;

3. The terminal vessels have the same radius R_{Tem} ($R_{\mathrm{Tem}} = 10\mu m$ in this work), thus the total number of terminal vessels is $N = \left\lceil \left(\frac{R_0}{R_{\mathrm{T}}}\right)^{\xi} \right\rceil$, where $\lceil x \rceil$ is the ceiling function of x;

4. For a given vessel i with $N_i (\geq 2)$ downstream terminal vessels, a random integer s_i is generated uniformly in $[1, N_i]$. Then, s_i terminal vessels are assigned to the left daughter vessel and the remaining ones are assigned to the right daughter vessel;

5. The radius of vessel i is $R_i = N_i^{1/\xi} R_{\mathrm{Tem}}$. The length-to-radius ratio of all vessels is the same as the structured tree, i.e., $L_r = 50$.

First, we compare the time profiles and phase profiles of the blood pressure and flow rate with the ST model and the random tree model (Fig. 1). The mean value of the blood pressure obtained with the flow input of the random tree is slightly smaller than that of the ST. On the other hand, the mean value of the blood flow rate obtained with the pulse pressure input of the random tree is slightly larger than that of the ST. The time profiles and the phase profiles of the blood pressure and flow rate with the two models are very similar to each other. The difference is mainly caused by the difference of the total resistance between the two models. Intuitively, the random tree bifurcates more "symmetrically" than the ST model. Thus the random tree has a smaller total resistance than the ST model.

The comparisons of impedance, kernel function, and the time profiles of blood pressure and flow rate in the case of a single artery among the random tree model, the corresponding WK model, and the CWK model are shown in Figs. 2-4. The results show that the modeling error induced by the WK and CWK models decreases when TR decreases and the CWK model gives rise to a smaller modeling error than the

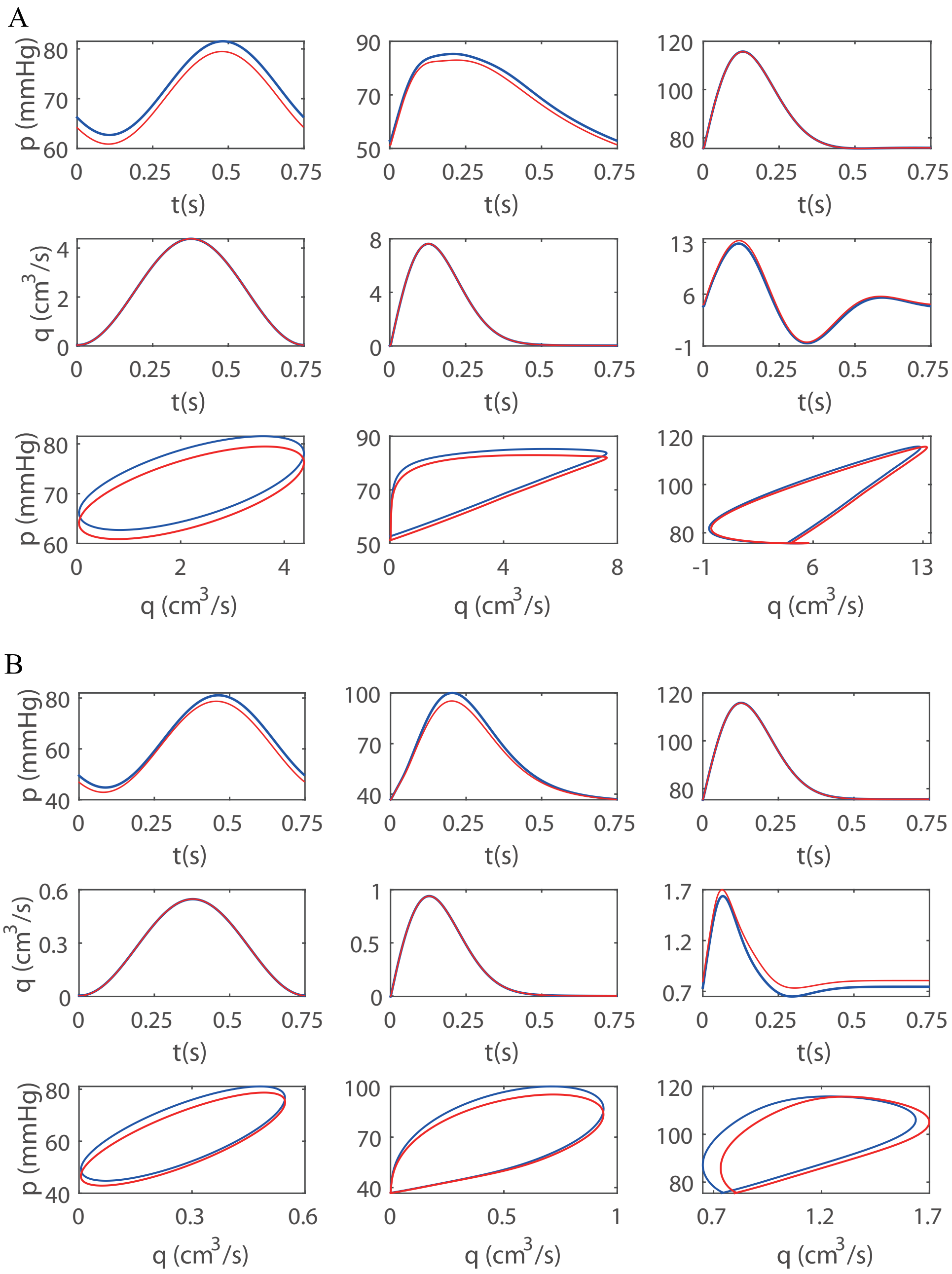

Figure 1. Time profiles and phase profiles of the blood pressure and flow rate at the mid-point of a single artery. The unstressed radii for Figure A and B are 0.26 cm and 0.13 cm, respectively. The left, middle, and right panels are obtained with the sinusoidal flow input, the pulsatile flow input, and the pulsatile pressure input, respectively. The blue and red lines correspond to the results obtained with the ST and random tree models, respectively.

WK model. The results are similar to those when the impedance of the ST model is used as reference.

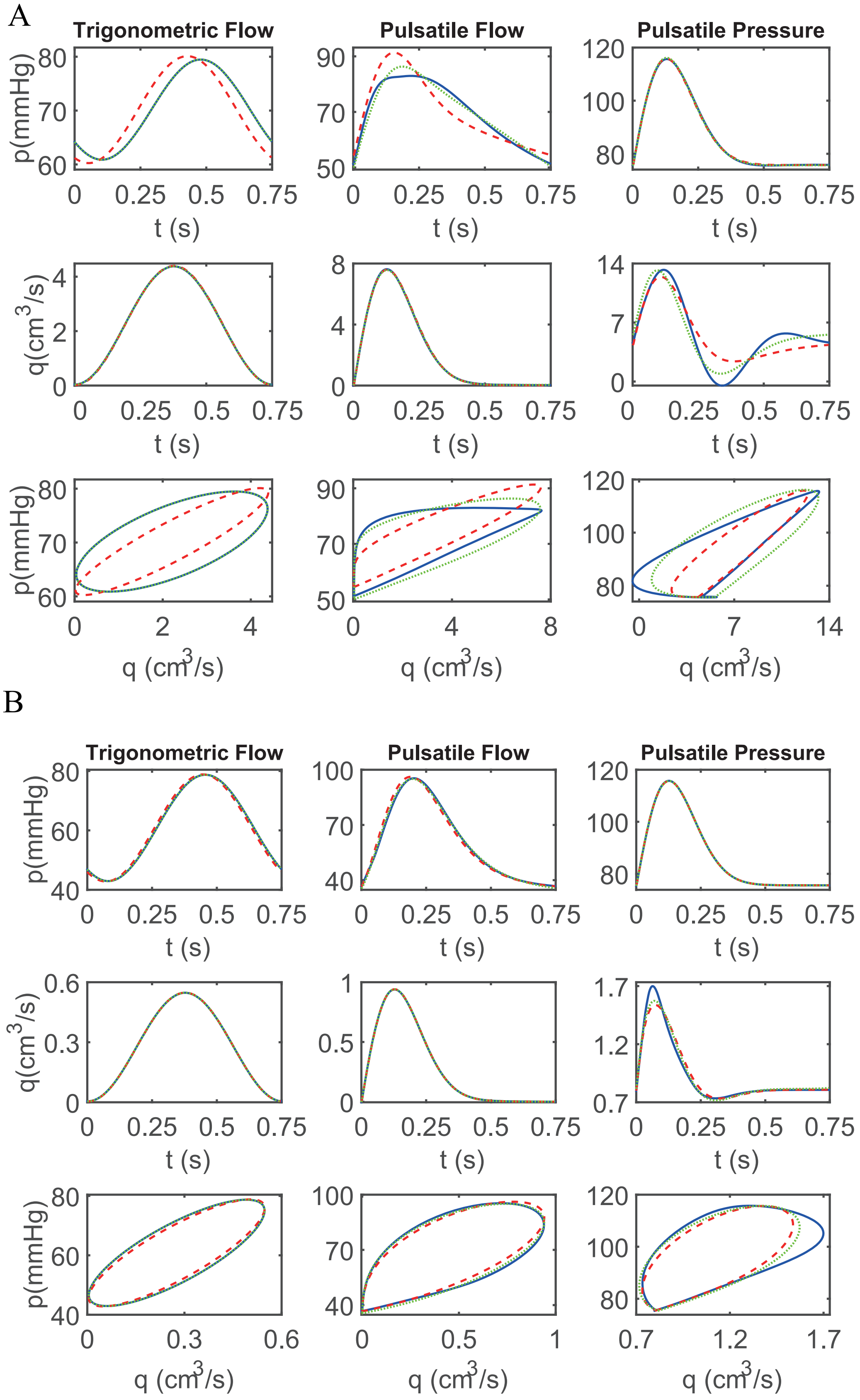

Figure 2. Time profiles and phase profiles of the blood pressure and flow rate at the mid-point of a single artery. The unstressed radii for Figure A and B are 0.26 *cm* and 0.13 *cm*, respectively. The left, middle, and right panels are obtained with the sinusoidal flow input, the pulsatile flow input, and the pulsatile pressure input, respectively. The blue, green, and red lines correspond to the results obtained with the random tree, CWK, and WK models, respectively.

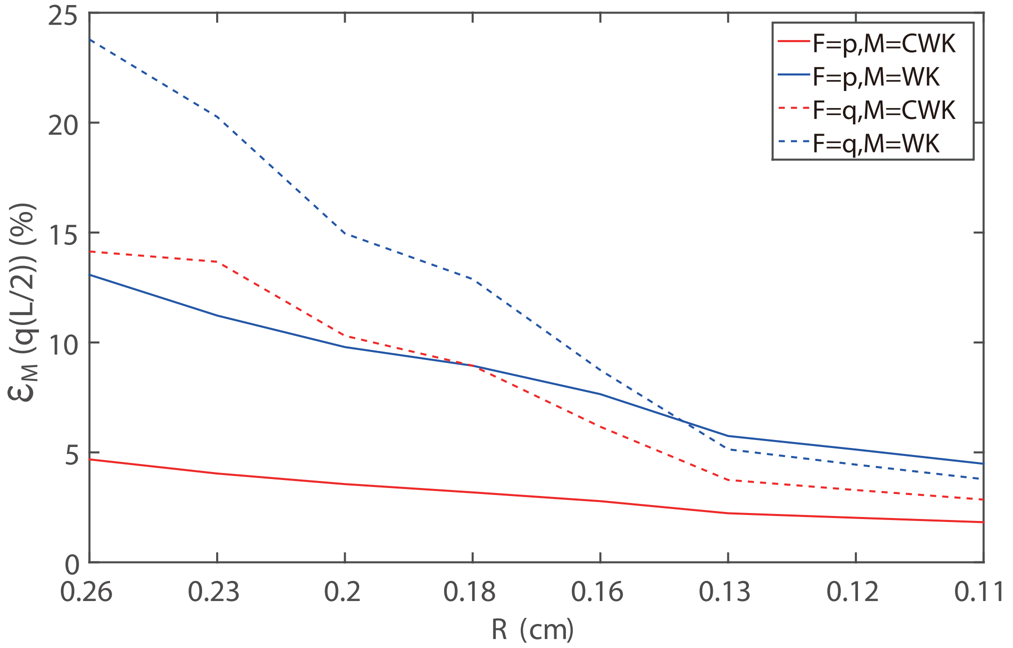

Figure 3. Modeling error induced by the WK and CWK models at the midpoint of a single artery. The x-axis is the unstressed radius of the single vessel. We compute the modeling errors of the blood pressure (red) and flow rate (blue) induced by the WK (dashed) and CWK (solid) model with the results using the random tree model as the reference in the case of a single artery.

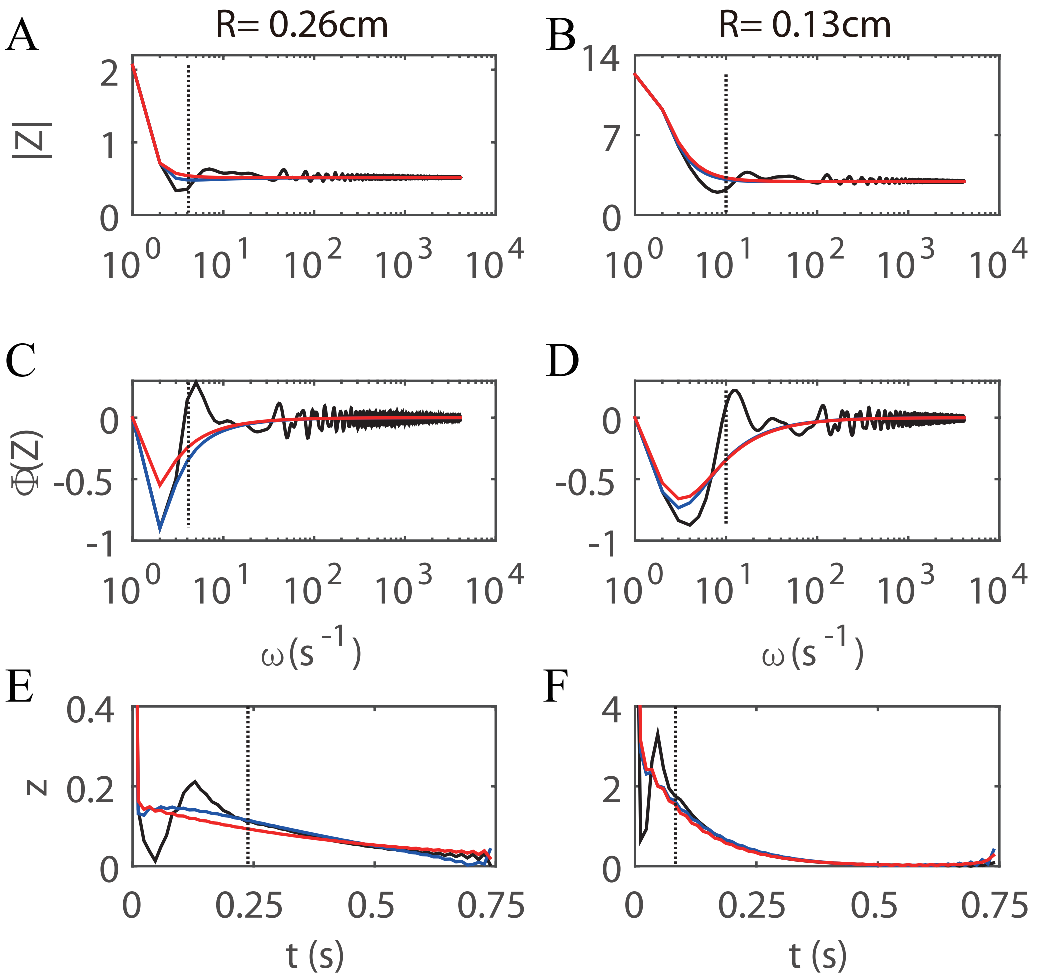

Figure 4. The impedance and kernel function obtained from the random tree model and from the corresponding WK and CWK models. The root-vessel radii are $R = 0.26cm$ and $R = 0.13cm$ for the left and the right panels, respectively, as marked on the top of the panels. The units of magnitude (A and B), phase (C and D) of the impedance, and the kernel function (E and F) are 10^4 $gcm^{-4}s^{-1}$, rad, and 10^5 $gcm^{-4}s^{-1}$, respectively. The vertical dotted line refers to the characteristic frequency in A-D and the corresponding characteristic time in E and F. The black, blue, and red lines correspond to the results obtained with the random tree, CWK, and WK models, respectively.
