# Supplementary figures and images for: Outflow Boundary Conditions for Blood Flow in Arterial Trees
Source: PLoS One. 2015 May 22;10(5):e0128597. doi: 10.1371/journal.pone.0128597 (PMC4441455; doi:10.1371/journal.pone.0128597)

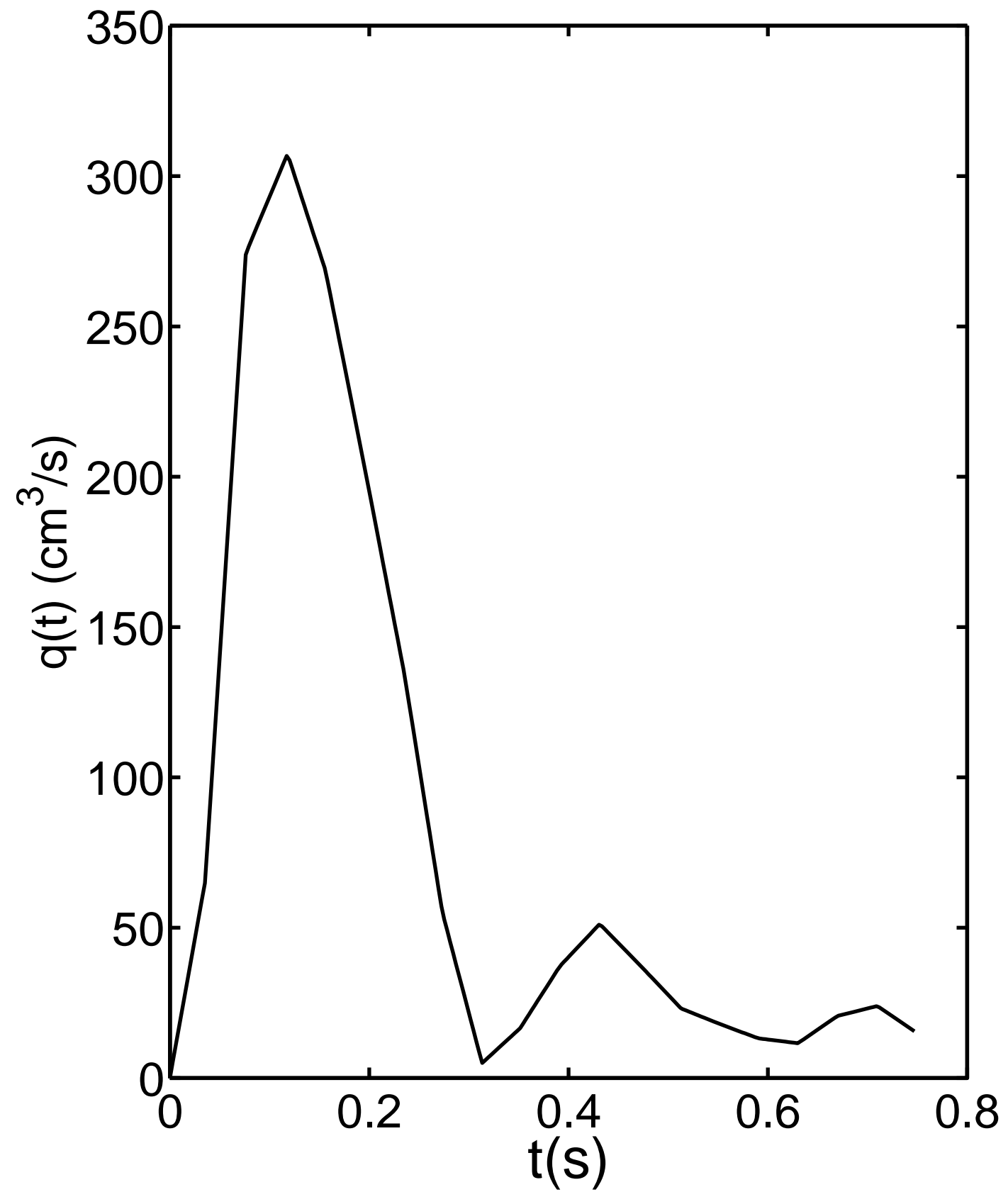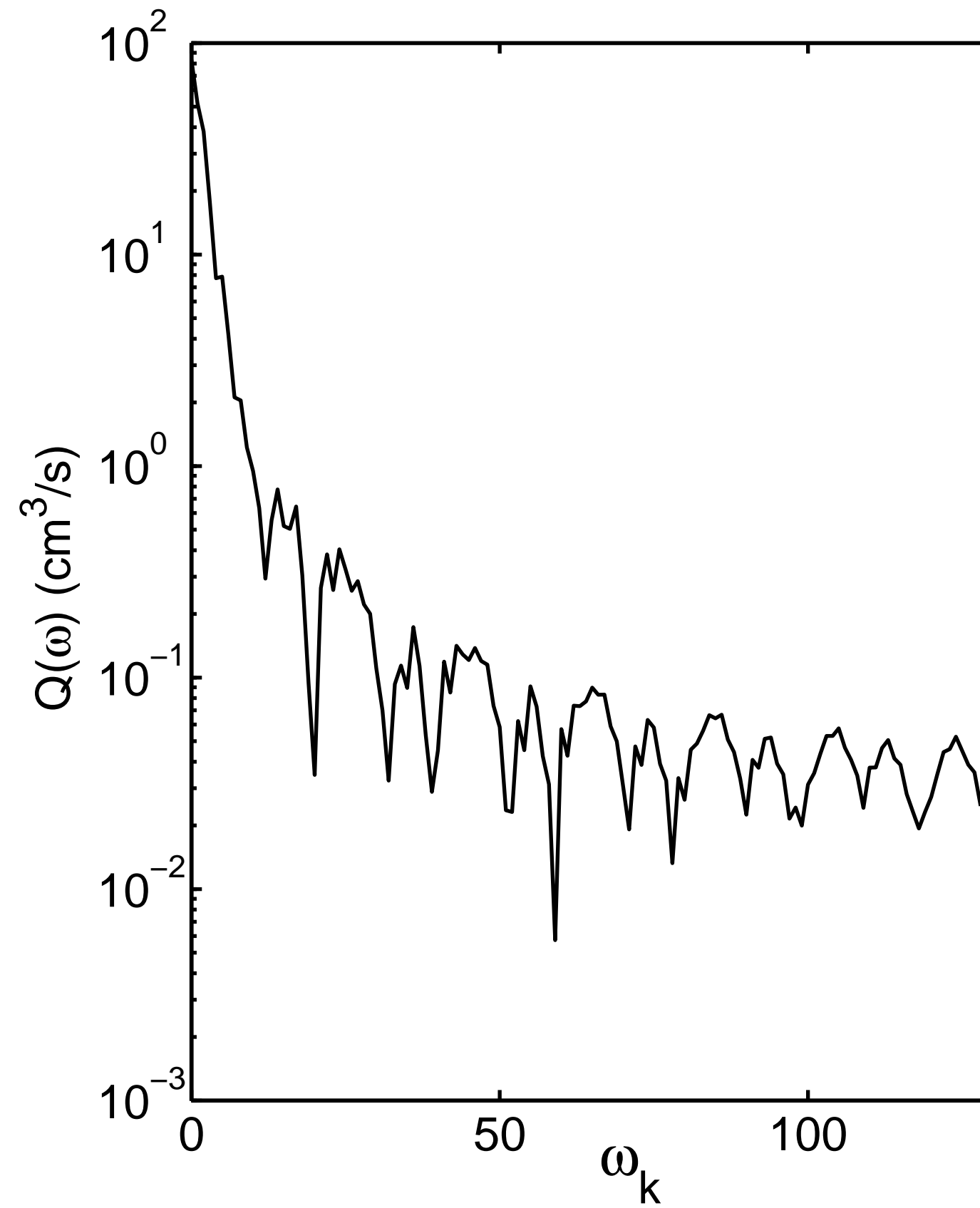

Supplement: S1 Fig — ω k in the x-axis of the right panel is ω k = kω 0. The blood flow rate is measured by an automated contour tracing method in the work of Ref. [56] (PDF) [file pone.0128597.s004.pdf]
